# An Investigation of WO_3_/V_2_O_5_/TiO_2_ Catalysts: Effects of WO_3_ on Morphology, Thermal Stability, and Activity for the Catalytic Oxidation of Dimethyl Sulfide

**DOI:** 10.3390/molecules30112436

**Published:** 2025-06-02

**Authors:** Gaytri Sharma, Endalkachew Sahle-Demessie, Catherine B. Almquist

**Affiliations:** 1 Chemical, Paper, Biomedical Engineering Department, Miami University, Oxford, OH 45056, USA; gaytrisharma@gmail.com; 2 US Environmental Protection Agency, Cincinnati, OH 45220, USA; sahle-demessie.endalkachew@epa.gov

**Keywords:** WO_3_/V_2_O_5_/TiO_2_ catalysts, morphology, DMS oxidation

## Abstract

WO_3_/V_2_O_5_/TiO_2_ (W_x_V_5_TiO_2_) catalysts were prepared via a wet incipient method with a V/Ti mass ratio = 0.05 and a W/Ti mass ratio varying from 0 to 0.10. The catalysts were calcined in air for 24 h at temperatures of 400 °C, 500 °C, 550 °C, and 600 °C. The presence of WO_3_ on W_x_V_5_TiO_2_ catalysts inhibits morphological and crystal structure transformations as the calcination temperature increases from 400 °C to 600 °C. The results of this study give evidence that the active component of the catalyst is V on anatase TiO_2_. Therefore, the incorporation of WO_3_ onto an anatase TiO_2_ support widens the temperature range at which the WO_3_/V_2_O_5_/TiO_2_ catalyst maintains the anatase crystal structure and, hence, the performance of the catalyst. The catalytic oxidation of dimethyl sulfide (DMS) was used as a probe reaction to evaluate catalytic activity. The results indicate that WO_3_/V_2_O_5_/TiO_2_ catalysts are capable of effectively oxidizing DMS at relatively low reaction temperatures (250 °C), even under conditions of an elevated DMS concentration in air (1.6 vol%).

## 1. Introduction

Catalytic oxidation is a widely applied technique for the abatement of volatile organic compounds (VOCs) from gaseous emissions streams, particularly in industry and environmental applications. This process involves the use of heterogeneous catalysts to facilitate the oxidation of VOCs at significantly lower temperatures than conventional thermal incineration, thereby reducing overall energy consumption and operational costs. Among its key advantages are high destruction efficiencies, minimal formation of secondary pollutants, and improved control over reaction conditions.

However, a primary limitation of catalytic oxidation is the potential for catalyst deactivation, which can compromise long-term process efficiency and reliability. Catalyst deactivation may result from exposure to elevated temperatures [1,2,3,4], which can lead to sintering or structural degradation of the active catalytic phase. In addition, the presence of compounds such as sulfur-containing species [3,5,6,7], chlorine, phosphorus, and alkali metals [8,9,10,11] can deactivate catalysts. These interactions can block active sites or alter the catalyst’s chemical composition, thereby reducing its catalytic activity and selectivity.

To maintain sustained catalytic performance, strategies such as catalyst regeneration, protective pretreatment of gas streams, and the development of more robust catalyst formulations are actively being investigated [12,13,14,15]. The selection of catalyst support materials and promoters also plays a critical role in enhancing resistance to poisoning and thermal degradation, thus extending catalyst lifespan under harsh operating conditions.

In the present study, the effects of the WO_3_ concentration on the thermal stability, morphology, and catalytic activity of WO_3_/V_2_O_5_/TiO_2_ catalysts were investigated for the oxidation of dimethyl sulfide (DMS). WO_3_/V_2_O_5_/TiO_2_ catalysts have been used commercially as a selective catalytic reduction (SCR) catalyst for de-NO_x_ applications [16,17] and have been investigated extensively [12,14,15,16,17,18]. However, these catalysts have also been found to be active for the oxidation of VOCs [18,19,20,21,22,23,24,25,26,27,28,29,30,31,32,33], including dioxins and chlorinated hydrocarbons [21,22], aromatics [23,24,25,26,27], and methanol [28,29,30,31,32,33]. Based upon a review of the literature, the vanadium species in these catalysts are active for both de-NO_x_ applications and for the oxidation of VOCs [16,17,18]. The addition of WO_3_ to the catalysts makes them less prone to thermal deactivation, and it inhibits the oxidation of SO_2_, making the catalysts more resistant to fouling by sulfur and alkali metals [16,17].

This paper makes two contributions to the scientific literature regarding this catalyst system: (1) it presents clear morphological changes in the catalyst in SEM images, with changes in the calcination temperature and W loading on WO_3_/V_2_O_5_/TiO_2_ catalysts; and (2) the coverage of vanadia species per unit surface area on anatase TiO_2_ significantly contributes to the active catalyst, whereas the surface area and WO_3_ have relatively minor roles in the catalytic activity of WO_3_/V_2_O_5_/TiO_2_ catalysts for the oxidation of dimethyl sulfide.

The motivation behind this study was to investigate the feasibility of using WO_3_/V_2_O_5_/TiO_2_ catalysts for treating non-condensable gases at their point of generation in a Kraft pulp and paper mill for either emission control or waste-to-value-added products. This motivation is shared by other researchers as well [34,35,36,37], who have researched methods to utilize and/or treat the waste gases from pulp and paper mills. Non-condensable gases often contain up to 5 vol% reduced sulfur compounds, with the predominant ones being dimethyl sulfide and methyl mercaptan [38]. Dimethyl sulfide (DMS) concentrations in pulp mill emissions can vary significantly based on the specific process stage, the equipment used, and whether emission control systems are in place. For instance, at evaporator vents prior to the scrubber, DMS concentrations can reach approximately 48,000 ppm (4.8%). However, the implementation of wet scrubbers can reduce this concentration to around 2500 ppm (0.25%) [39,40]. Currently, non-condensable gases are often directed over long distances through (sometimes miles of) piping to the mill’s recovery boiler, where the gases are burned, and the pulping chemicals are recovered. This practice is energy intensive, and it often burdens and/or limits the capacity of the recovery boiler at the mill.

**Figure 1:**
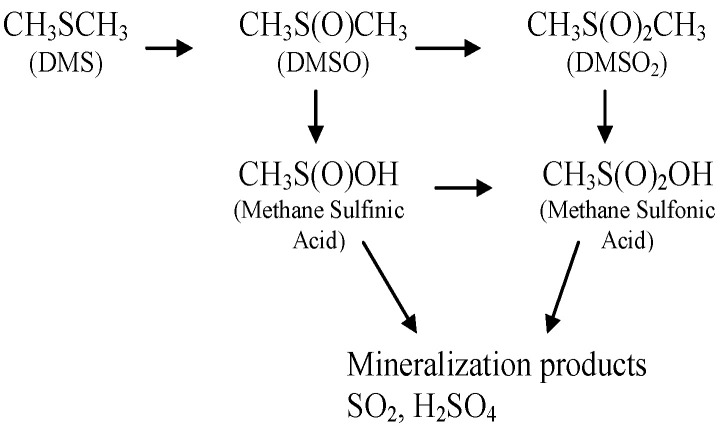
DMS oxidation sulfur-containing products and by-products.

The oxidation of dimethyl sulfide has been investigated by several researchers, in the atmosphere [41], with ozone and ozone-enhanced catalysis [42,43,44,45,46], with oxygen and catalysts [47,48], and with photocatalysts [49,50,51]. The predominant by-products of dimethyl sulfide (DMS) oxidation include dimethyl sulfoxide (DMSO), dimethyl sulfone (DMSO_2_), methane sulfinic acid, and methane sulfonic acid. In addition, complete mineralization products of sulfur have been observed, including SO_2_ and H_2_SO_4_. Possible mechanisms for vapor-phase oxidation of dimethyl sulfide are cited in the literature [41,42,48,49,50,51]. Sulfur-containing oxidation products of DMS are shown in Figure 1.

The goals of this study were to investigate the relative activities of the V/TiO_2_ catalysts as functions of WO_3_ loading and the calcination temperature. We used DMS as a probe molecule for this purpose. While a thorough investigation on industry-relevant conditions is recommended for future studies, these data were not acquired in this study.

## 2. Results

### 2.1. Catalyst Characterization

Table 1 summarizes the characteristics of the catalysts, including the BET surface area and average pore size (calculated based on nitrogen adsorption and desorption at −196 °C), the anatase crystal size and percent anatase calculated from XRD diffractograms, and the coverage of V on the surface area of the catalyst, depicted in this study as the number of monolayers of V on TiO_2_. For this study, the monolayer coverage was approximated to be 10 V atoms/nm^2^ surface area based upon the review written by Grzybowka-Swierkosz [18]. However, the monolayer coverage of V on TiO_2_ has been reported to be as low as 4.4 V atoms/nm^2^ [52] and approximately 8 V atoms/nm^2^ by other researchers [31]. Equations (1) and (2) were used to define monolayers of V in this study.

(1) $$atoms\_V/{nm}^{2}=\frac{\frac{x_{V}}{{MW}_{V}}\times (6.02\times 10^{23})}{SSA\times (1\times 10^{18})}$$

(2) $$monolayers\_V=\frac{atoms\_V/{nm}^{2}}{10\;atoms\_V/{nm}^{2}}$$

Here, x_V_ is the mass fraction of V on the catalyst (g V/g catalyst), MW_V_ is the atomic mass of V (g V/mole), 6.02 × 10^23^ is Avogadro’s number (atoms/mole), SSA is the specific surface area of the catalyst (m^2^/g catalyst), and 1 × 10^18^ is the conversion from m^2^ to nm^2^.

The anatase phase of TiO_2_ was predominant in all the samples calcined at 400 °C and 500 °C. However, further increases in the calcination temperature produced significant changes in the crystal structure of the TiO_2_ support, with evidence of anatase-to-rutile phase transformation observed in certain catalysts. Two observations were made: (1) the presence of V on the catalyst surface significantly lowers the temperature at which sintering and anatase-to-rutile phase transformation occurs; and (2) the presence of WO_3_ inhibits changes in the crystal structure in W_x_V_5_TiO_2_ catalysts as the calcination temperature increases. The first observation is supported by several studies [33,54,55,56,57,58,59]. Balikdjian et al. [57] referenced the “sintering-induced phase transition” model proposed by Amores, et al. [55] that suggests surface V species promote sintering, even at low concentrations. The sintering process raises the catalyst surface temperature, facilitating rutile nucleation. Huang et al. [56] demonstrated that the presence of V_2_O_5_ significantly enhanced the densification of Sr_0.4_Ba_0.6_Nb_2_O_6_ ceramics, reducing the sintering temperature from 1425 °C to 1100 °C. Although the calcination temperatures in this study were much lower than those in the study by Huang et al. [56], it is analogous that V_2_O_5_ spreads out on the TiO_2_ surface, promoting its densification. Balikdjian et al. provided experimental evidence in support of the sintering-induced phase transition mechanism [57]. Sintering promotes surface smoothing, particle joining, and pore rounding [60]. Since V_2_O_5_ has a much higher vapor pressure than either TiO_2_ or WO_3_, sublimation and vapor transport of V species to surfaces of lower vapor pressure may be a mechanism of initial sintering for these catalysts as the calcination temperature increases. Surface V species likely enhance the particle boundary mobility, which will allow the particles to be drawn closer together and rearrange into a denser configuration (e.g., anatase to rutile TiO_2_). In addition, V_2_O_5_ can be considered a flux material that has a melting point of approximately 690 °C. Fluxes enhance atomic diffusion during solid-state sintering, thus accelerating phase transitions [60].

The second observation, the inhibition effect of WO_3_ on V_2_O_5_/TiO_2_ catalyst sintering, is also supported by many other researchers [2,3,7,16,17,33,54,61]. Reed [60] states that grain growth is inhibited when a dopant exceeds the solubility limit and a well-dispersed second phase exists at the grain boundaries. Thus, in the W_x_V_5_TiO_2_ catalysts, the WO_3_ would be relatively insoluble in TiO_2_ at the calcination temperatures used in this study. In addition, it would be well dispersed on the catalyst surface. Therefore, its presence on the surface of the catalyst would inhibit densification, hence, conversion from anatase to rutile TiO_2_. This is clearly shown in the SEM images shown in Figure S1, which depicts the morphologies of the catalysts calcined at 600 °C. The spherical anatase TiO_2_ features are preserved to a greater extent as WO_3_ loading increases. 

**Figure 2:**
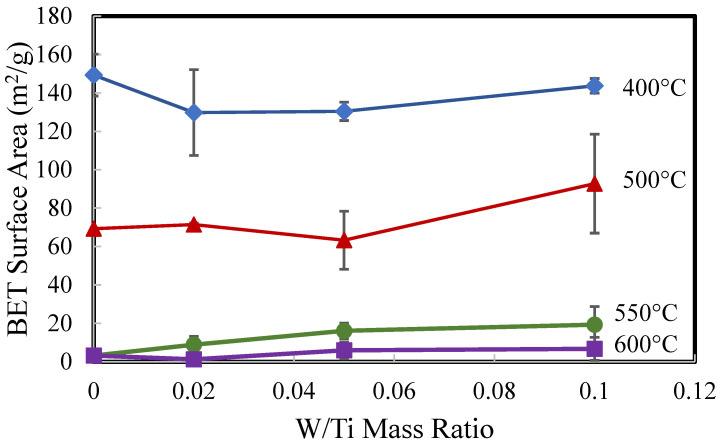
BET surface area of W_x_V_5_TiO_2_ catalysts as functions of the calcination temperature and W/Ti mass ratio. (x = 0, 2, 5, and 10, corresponding to W/Ti mass ratios = 0, 0.02, 0.05, and 0.1). All catalysts have a V/Ti mass ratio = 0.05.

The changes in BET surface area for W_x_V_5_TiO_2_ catalysts as functions of W/Ti mass ratios and the calcination temperature are provided in Figure 2. The specific surface area decreases significantly with the calcination temperature for all catalyst compositions. Therefore, sintering of the catalysts occurs with and without the addition of WO_3_ as the calcination temperature increases from 400 °C to 600 °C. This is also shown in TEM images in Figures S2–S5, where catalyst particle sizes significantly increase with calcination temperature from 400 °C to 500 °C. The reduction in surface area is the result of surface smoothing of particles, neck growth between adjacent particles, and shrinkage and/or blocking of pores. However, at each calcination temperature, the BET surface area generally increases with W/Ti mass ratios from 0.02 to 0.10.

**Figure 3:**
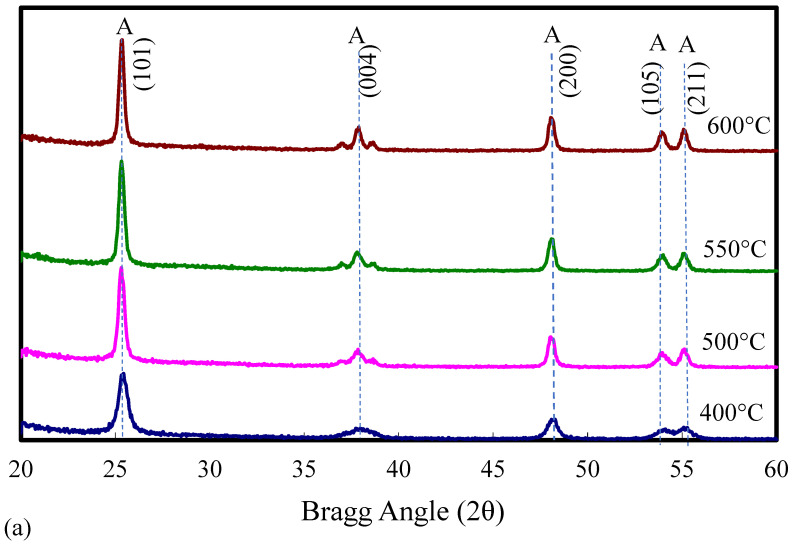

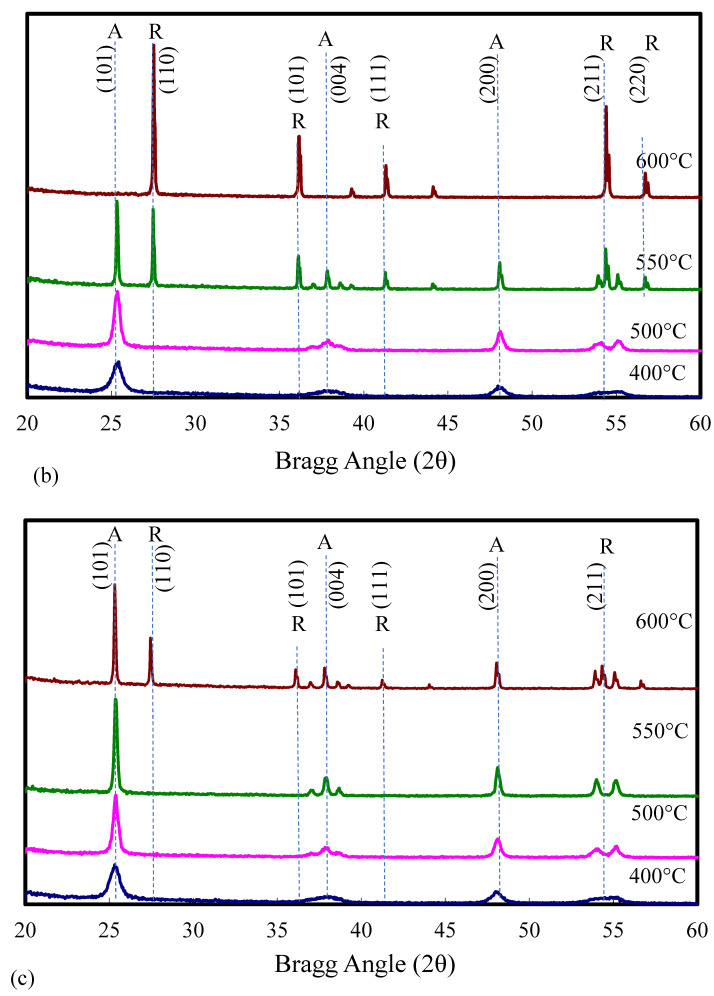

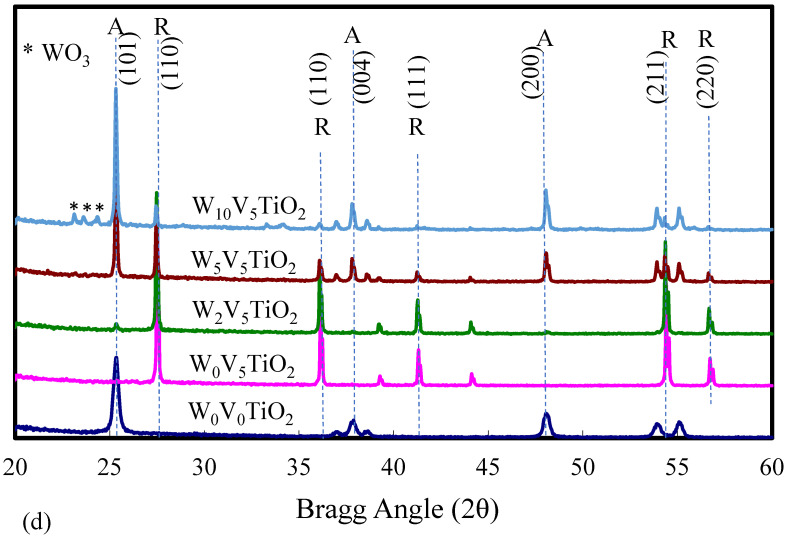
XRD diffractograms of catalysts after calcination at temperatures from 400 °C to 600 °C. (**a**) Pure TiO_2_, (**b**) W_0_V_5_TiO_2_, (**c**) W_5_V_5_TiO_2_, and (**d**) W_x_V_5_TiO_2_ catalysts calcined at 600 °C, where x = 0, 2, 5, and 10 corresponding to W/Ti mass ratios = 0, 0.02, 0.05, and 0.1. A = Anatase TiO_2_; R = Rutile TiO_2_.

Figure 3a–d show the XRD diffractograms of the catalysts. Figure 3a shows the XRD diffractograms for pure ST01 TiO_2_ calcined at various temperatures. It was observed that the crystal structure of pure ST01 TiO_2_ was anatase at all calcination temperatures studied (400–600 °C). This is supported by other researchers, who reported that the anatase-to-rutile phase transformation of pure anatase TiO_2_ takes place in the temperature range of 700 °C to 900 °C [54,55,57,58,62].

Figure 3b shows the XRD diffractograms of W_0_V_5_TiO_2_ catalysts calcined at temperatures from 400 °C to 600 °C. Both anatase and rutile crystal phases of TiO_2_ are present following calcination at 550 °C, and a nearly complete phase transition to rutile occurred at 600 °C. This shows that V loading promotes sintering and phase transformation in TiO_2_. No crystalline V_2_O_5_ is observed on the XRD diffractograms. This provides evidence that the vanadia species are predominantly amorphous, and, if present, crystalline vanadia species are below the detection limit of XRD.

Figure 3c presents the XRD diffractograms of W_5_V_5_TiO_2_ catalysts as a function of the calcination temperature. The results indicate that the TiO_2_ remained predominantly in the anatase phase up to 550 °C, with the anatase phase still dominant even at 600 °C. These observations are consistent with previous reports [2,3,7,16,17,33,54,61], which suggests that WO_3_ inhibits the anatase-to-rutile phase transformations in TiO_2_. Since anatase is the more catalytically active polymorph of TiO_2_, the incorporation of WO_3_ effectively extends the thermal stability range of the catalyst, broadening its operational temperature window. Notably, no crystalline phases corresponding to V_2_O_5_ or WO_3_ were detected in the W_5_V_5_TiO_2_ catalysts’ XRD diffractograms, indicating either high dispersion or amorphous character of these species.

Figure 3d shows the XRD diffractograms of W_x_V_5_TiO_2_ catalysts after calcination in air at 600 °C, where x is varied from 0 to 10 (W/Ti mass ratio = 0 to 0.10). As the W/Ti mass ratio increases from 0 to 0.1, the anatase TiO_2_ support is remarkably preserved. W_0_V_5_TiO_2_ is nearly all rutile after calcination at 600 °C, while W_10_V_5_TiO_2_ is nearly all anatase under the same conditions. Although sintering of the catalysts occurs with W loading, as indicated by the reduction in BET surface area after calcination of the catalysts from 400 °C to 600 °C, W loading significantly inhibits the densification of the TiO_2_, and so the anatase crystal structure is preserved. Additionally, weak diffraction features characteristic of crystalline WO_3_ begin to appear in the XRD diffractograms of the W_10_V_5_TiO_2_ catalysts after calcination at 600 °C (20° < 2θ < 25°), suggesting partial crystallization of WO_3_ at higher loadings.

**Figure 4:**
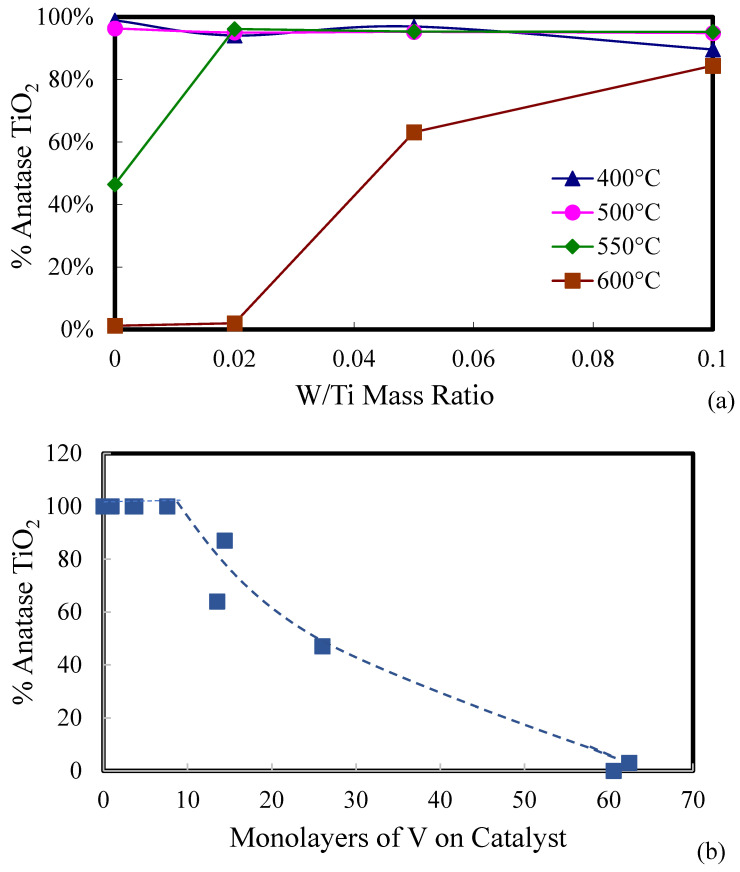
The percent anatase TiO_2_ in W_x_V_5_TiO_2_ catalysts (**a**) as functions of the calcination temperature and W/Ti mass ratio and (**b**) as a function of monolayers of V on the catalyst.

The fraction anatase in each catalyst was calculated using Equation (3), an equation that was reported in [53]. Figure 4a shows the anatase fraction in W_x_V_5_TiO_2_ catalysts as a function of the W/Ti mass ratio and calcination temperature. At calcination temperatures ≤ 500 °C, no significant effect of the W/Ti mass ratio is seen on the crystal structure of TiO_2_, since the crystal structure of TiO_2_ in all catalysts calcined at 400 °C and 500 °C is anatase. Therefore, the data points in Figure 4a for catalysts calcined at 400 °C and 500 °C overlap. However, after calcination at 550 °C and 600 °C, there is a clear role of WO_3_ as an inhibitor of TiO_2_ phase transformation from anatase to rutile.

(3) $$F_{anatase}=\frac{1}{1+1.26(I_{r}/I_{A})}$$

Here, *F_anatase_* = the fraction of anatase TiO_2_, *I_A_* = the intensity of the strongest anatase peak (2θ = 25.3), and *I_r_* = the intensity of the strongest rutile peak (2θ = 27.4).

Figure 4b shows the anatase fraction in W_x_V_5_TiO_2_ catalysts as a function of monolayers of V (as defined in Equations (1) and (2)) on the TiO_2_ support. As shown in Figure 4b, the anatase fraction decreases after approximately eight monolayers of V on TiO_2_ are reached.

**Figure 5:**
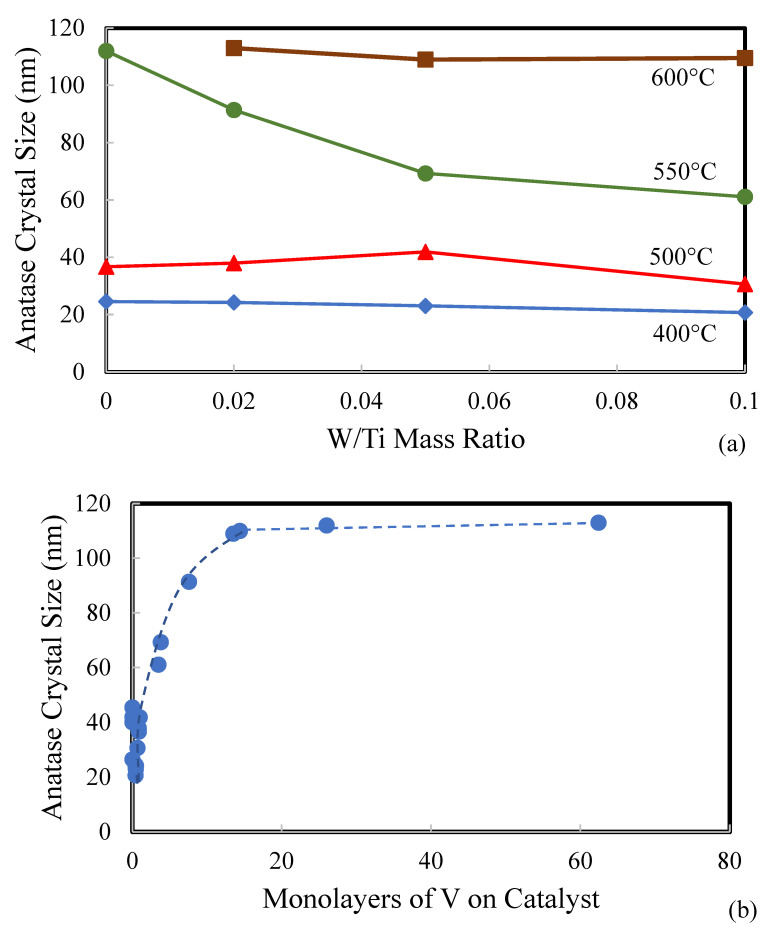
(**a**) Anatase crystal size in W_x_V_5_TiO_2_ catalysts as functions of the calcination temperature and W/Ti mass ratio and (**b**) as a function of monolayers of V on the catalyst.

The anatase crystal sizes in W_x_V_5_TiO_2_ catalysts were calculated by using the Debye–Scherrer equation (Equation (4)). As shown in Figure 5a, the anatase crystal size increases as the calcination temperature increases. There appears to be a critical crystal size of anatase at approximately 110 nm, above which TiO_2_ phase transformation occurs from anatase to rutile. This is also shown more clearly in Figure 5b, which correlates the anatase crystal size with the number of monolayer loadings of V on TiO_2_. After approximately 15 monolayers of V, the anatase crystal size reached a plateau at approximately 110 nm. An anatase crystal size plateau has been reported by other researchers who studied the anatase-to-rutile phase transition, including Perego et al. [62] who reported an anatase particle size plateau at approximately 40 nm, and Zhang et al. [63], who reported a plateau at approximately 80 nm. Additional support was found by Orendorz et al. [64], who reported anatase crystal sizes as large as 125 nm. Further sintering of these anatase crystals results in densification and phase transformation from anatase to rutile. Perego et al. [62] found that changes in the morphology of anatase TiO_2_ impacted the anatase-to-rutile transformation process by altering the rate of rutile nucleation at the interface between two anatase surfaces. Analogously, the addition of WO_3_ to the anatase TiO_2_ surfaces would change its surface morphology and, thus, inhibit the rutile nucleation process between two anatase surfaces.

(4) $$d_{p}=\frac{k\lambda }{B_{1/2}\mathrm{Cos}\theta }$$

Here, *d_p_* = the diameter of the anatase crystalline particle (nm). *k* = 0.94. *B*_1/2_ = the half height width of the predominant anatase peak (nm). λ = the wavelength of X-rays (1.54 Å). θ = the angle measured from the diffraction pattern.

**Figure 6:**
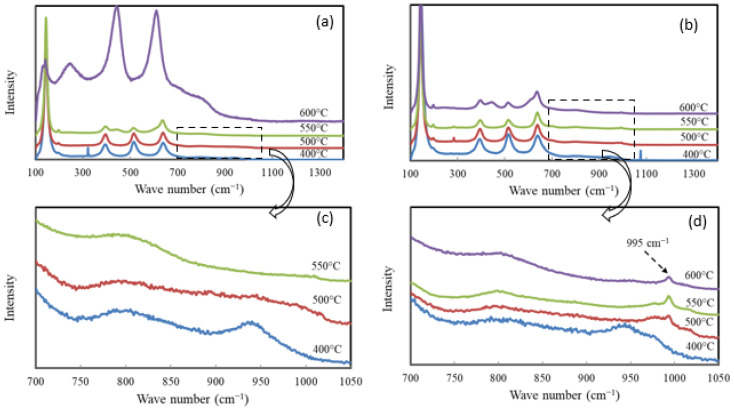
Raman spectra of (**a**) W_0_V_5_TiO_2_ catalysts and (**b**) W_5_V_5_TiO_2_ catalysts calcined at temperatures from 400 °C to 600 °C; enlarged views of the Raman spectra of (**c**) W_0_V_5_TiO_2_ catalysts and (**d**) W_5_V_5_TiO_2_ catalysts calcined at temperatures from 400 °C to 600 °C.

Figure 6a,b show the Raman spectra of selected catalysts, providing complementary information to the XRD data. Raman spectroscopy is particularly useful for detecting the presence of V and W species at low concentrations. Figure 6a illustrates the effect of the calcination temperature on W_0_V_5_TiO_2_ catalysts, and Figure 6b shows the same for W_5_V_5_TiO_2_ catalysts. In each figure, the spectral region between 700 cm^−1^ and 1050 cm^−1^ is magnified to highlight signals associated with vanadia species.

The Raman spectra data support the XRD data with respect to anatase-to-rutile transformation. Characteristic anatase TiO_2_ peaks at 144, 393, 151, and 638 cm^−1^ [59] are predominant in all spectra shown in both Figure 6a,b with one exception; the spectra for the W_0_V_5_TiO_2_ catalyst after calcination at 600 °C shows predominantly rutile TiO_2_ features (142, 247, 441, and 610 cm^−1^ [59]). Minor rutile features are also observed in the W_0_V_5_TiO_2_ catalyst calcined at 550 °C and in the W_5_V_5_TiO_2_ catalyst calcined at 600 °C. These observations in the Raman spectra support the XRD data that show that the addition of WO_3_ to the catalysts inhibit the anatase-to-rutile TiO_2_ transformation.

The enlarged sections of the Raman spectra (Figure 6c,d) show two broad bands near 800 cm^−1^ and 950 cm^−1^, and one sharp band appearing at 995 cm^−1^. The broad band near 800 cm^−1^ could be a minor peak characteristic of anatase TiO_2_, since it is also observed in TiO_2_ samples containing neither V nor W (Figure S6). The broad band near 950 cm^−1^ may be attributed to polymeric vanadate (V=O) groups [65,66,67]. The peak at 995 cm^−1^, which is observed in the W_5_V_5_TiO_2_ catalysts calcined at temperatures of 500 °C and higher can be attributed to the stretching vibration mode of V=O groups in crystalline V_2_O_5_ [65,66]. Therefore, the Raman data give evidence of V_2_O_5_ crystallization, and the presence of WO_3_ promotes it. The discrepancy between Raman and XRD results with respect to crystalline V_2_O_5_ could arise from the fact that the detection limits of Raman spectroscopy are lower than those for XRD [68,69]. This was reported by Curcio et al. [69], who stated that Raman spectra are very sensitive, even to slight structural modifications that are below the detection limit of conventional characterization techniques, such as X-ray diffraction. Raman spectroscopy excels at surface-sensitive, molecular-level analysis and is useful for nanoscale or single particle measurements, whereas XRD is excellent for determining crystalline phase composition and crystallite size. However, the detection limits of XRD are in the range of 0.1 wt% to 1 wt% per phase [70].

The morphologies of the W_x_V_5_TiO_2_ catalysts were investigated via scanning electron microscopy (SEM), and the compositions of various features on the catalysts were investigated using EDX (Figures S7–S16). The phases and elemental composition of features observed in the SEM micrographs were supported with EDX, XRD, and Raman analyses.

**Figure 7:**
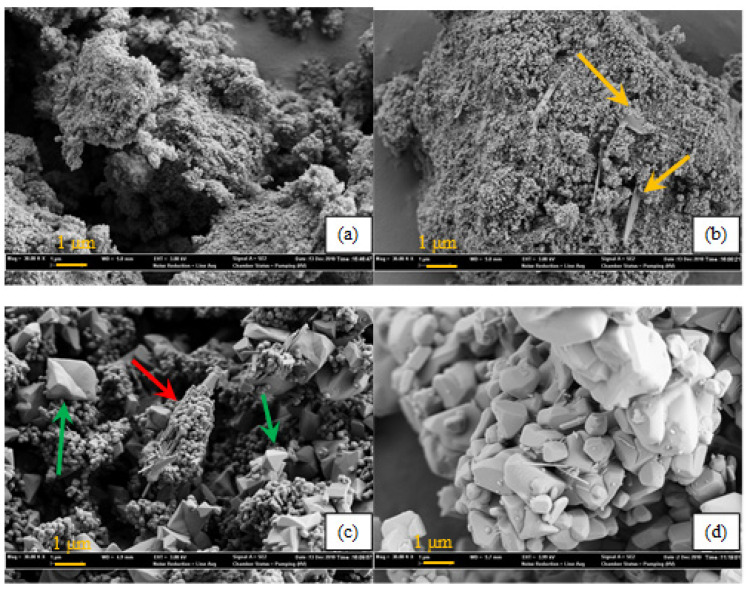
SEM images (30,000 magnification) of W_0_V_5_TiO_2_ catalysts calcined at different temperatures in air for 24 h. (**a**) 400 °C; (**b**) 500 °C; (**c**) 550 °C; (**d**) 600 °C. Orange arrows: Titania vanadia species. Red and Green arrows: Rutile TiO_2_ structures.

Figure 7 shows the influence of the calcination temperature on the morphology of the W_0_V_5_TiO_2_. Noted are two key observations: (1) features containing predominantly V (per EDX analyses) start appearing even after calcination at 500 °C, and (2) the presence of V promotes sintering and phase transformation of small (<100 nm), spherical anatase crystals (Figure S7) to large (>1 um) rutile structures. Ribbon-like structures appear after calcination at 500 °C, as shown by the yellow arrows, as evidenced in Figure S8. Tetrahedron-shaped rutile TiO_2_ features (Figure S9) appear after calcination at 550 °C (green arrows), as do clusters of rod-like structures (red arrows); these features coalesce and sinter to form large (>1 um) rutile structures after calcination at 600 °C. The structural modification of TiO_2_ to the rutile phase is highly undesirable, as it leads to a significant decrease in the surface area and loss of catalytic activity.

Structures containing predominantly V are distinct temperature-dependent morphological structures, such as the ribbon-like structure after calcination at 500 °C. The transition of these V-containing structures as a function of temperature are consistent with the observations reported by Loffler et al. (2011) [71], who used SEM micrographs to investigate the formation of V nanocrystals as a function of the calcination temperature. Their results support the observations made in Figure 7. Loffler et al. [71] investigated the formation of vanadium oxide nanocrystals as a function of the calcination temperature and found three distinct growth regimes: vanadium oxide nanowires (<500 nm) were formed at temperatures < 450 °C, sheets (~1 um wide) were formed at temperatures between 450 °C and 600 °C, and rods (~10 um long) were formed at temperatures greater than 650 °C. Similar morphological features were observed in the catalyst samples analyzed in this study, supporting the trends noted in Figure 7.

**Figure 8:**
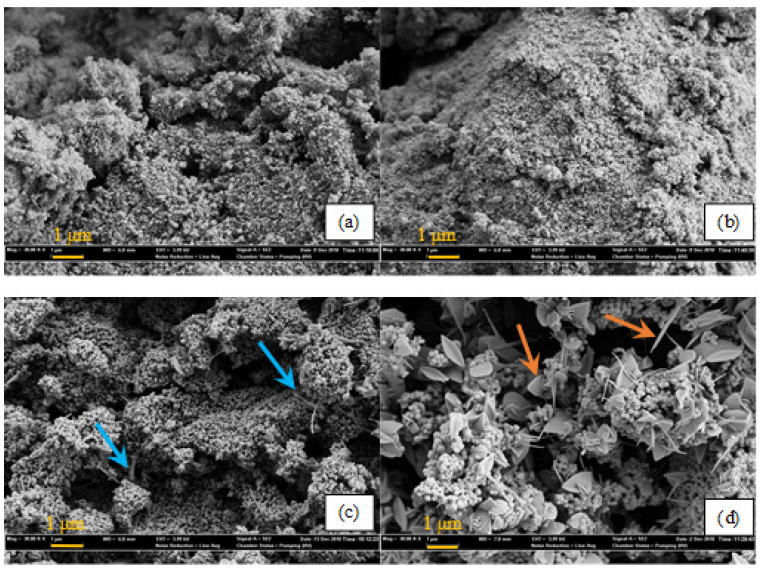
SEM images (30,000 magnification) of W_5_V_5_TiO_2_ catalysts calcined at different temperatures for 24 h. (**a**) 400 °C; (**b**) 500 °C; (**c**) 550 °C; (**d**) 600 °C. Blue arrows: rod-like Rutile TiO_2_ structures. Orange arrows: plate-like Rutile TiO_2_ structures.

Figure 8a–d show the SEM images of the W_5_V_5_TiO_2_ catalysts, highlighting their morphological changes with an increasing calcination temperature. Needle- and rod-like V-structures are apparent after calcination at 550 °C (Figure 8c, blue arrows) in W_5_V_5_TiO_2_ catalysts, while the spherical anatase TiO_2_ particles (<100 nm) remain the dominant morphology even after calcination at 550 °C. Plate-like rutile features (orange arrows) appear after calcination at 600 °C, consistent with the onset of anatase-to-rutile phase transformation.

A comparison of Figure 7 and Figure 8 clearly demonstrates that the incorporation of WO_3_ on the catalysts inhibits the initial formation of V-containing ribbon-like structures. In addition, the presence of WO_3_ significantly affects the rutile crystal structure following phase transformation after calcination at 600 °C. In the absence of WO_3_, the rutile structures are notably large (>1 um), first appearing as tetrahedron-like structures at 550 °C and subsequently coalescing into boulder-like aggregates as the calcination temperature increases to 600 °C. However, in W_5_V_5_TiO_2_ catalysts, the rutile structures are more plate-like or even have a “butterfly”-like morphology, and the spherical anatase features, while much larger (~100 nm compared to ~20 nm), are still present. This observation indicates that WO_3_ both inhibits phase transformation of anatase to rutile and alters the crystal structure of rutile once it is formed. This is consistent with the findings of Cristallo et al. [72], who studied the morphological changes in TiO_2_ doped with V_2_O_5_ and with both V_2_O_5_ and WO_3_. They found that the rutile phase in catalysts containing WO_3_ grows in only two directions, resulting in plate-like rutile structures, and the (101) plane in rutile is the preferential growth plane [72].

### 2.2. Catalysis

**Figure 9:**
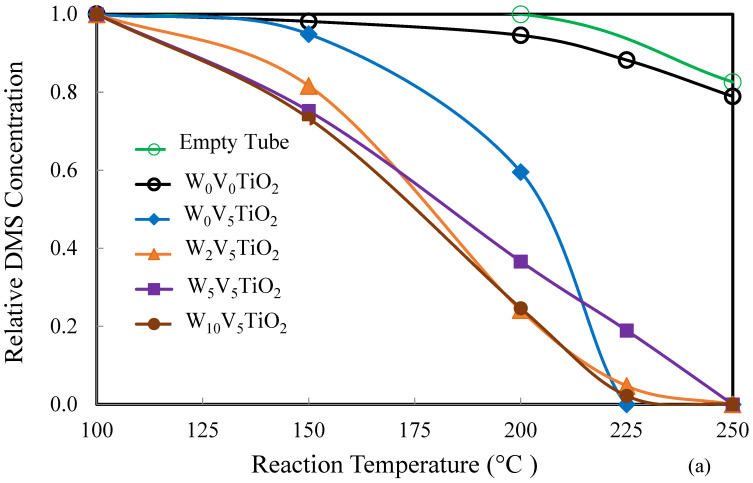

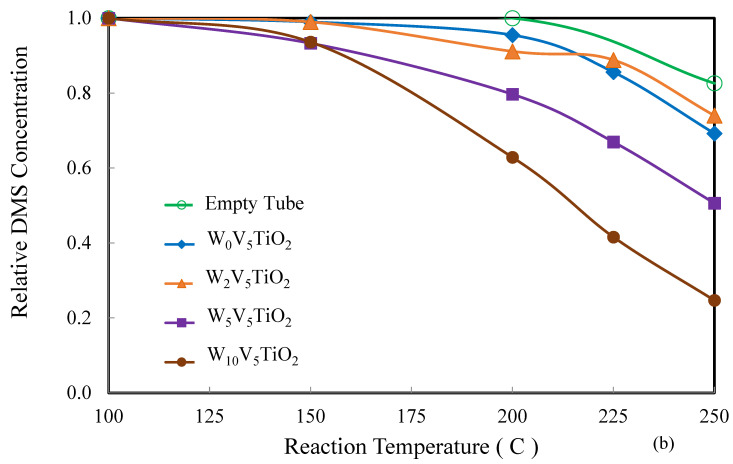
Degradation of DMS over W_x_V_5_TiO_2_ catalysts calcined at (**a**) 500 °C and (**b**) 600 °C.

The performance of the catalysts was assessed using the oxidation of dimethyl sulfide (DMS) as a relative measure of catalytic activity. Figure 9a,b show the effects of W/Ti mass ratio on DMS oxidation after the catalysts were calcined at 500 °C (Figure 9a) and 600 °C (Figure 9b). Figure 9a shows that all V-containing catalysts calcined at 500 °C can degrade > 95% of the DMS at a reaction temperature of 250 °C. In contrast, under identical conditions, DMS conversion was less than 20% in both the blank reactor (i.e., without catalyst) and when using pure TiO_2_ as the catalyst. This confirms that the presence of V on anatase TiO_2_ is responsible for the catalytic activity for the oxidation of DMS, and it implies that WO_3_ is not catalytically active for the oxidation of DMS. Figure 9b shows that catalysts containing a W/Ti mass ratio of 0.05 and 0.10, after calcination at 600 °C has activity for DMS oxidation greater than those with W/Ti mass ratios of 0 and 0.02. Since W loading inhibits phase transformation from anatase to rutile, the activity of the catalyst is preserved even after calcination at 600 °C in W_5_V_5_TiO_2_ and W_10_V_5_TiO_2_ catalysts.

**Figure 10:**
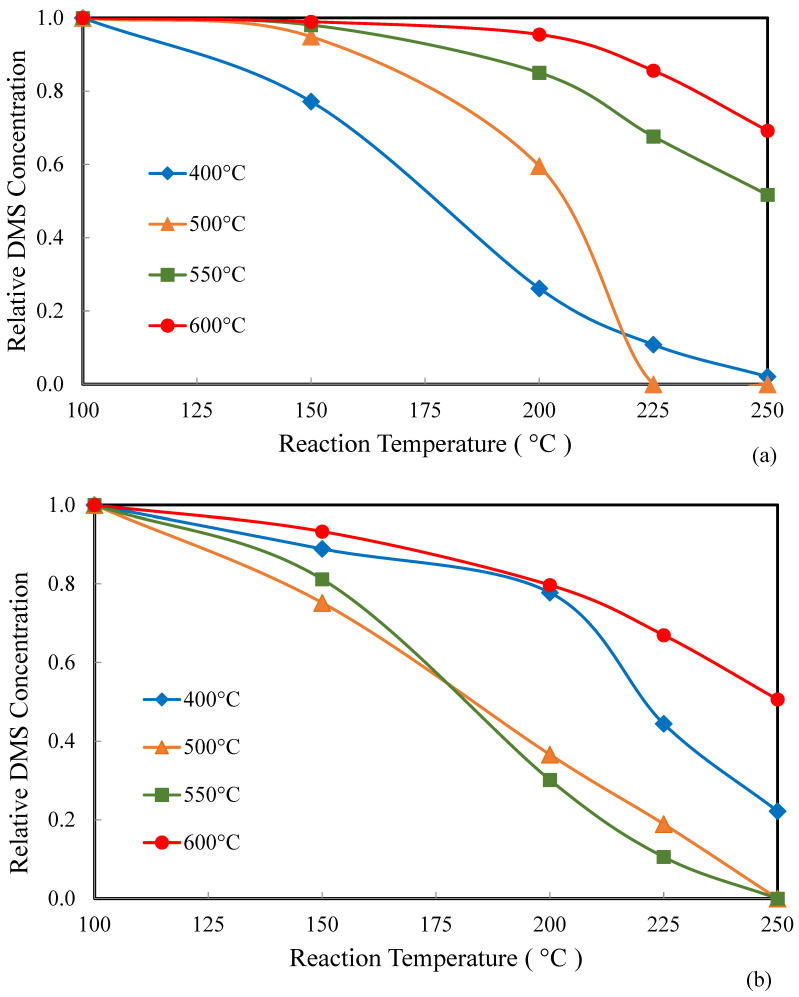
DMS catalytic oxidation over (**a**) W_0_V_5_TiO_2_ catalysts and (**b**) W_5_V_5_TiO_2_ catalysts calcined at temperatures ranging from 400 °C to 600 °C.

Figure 10a,b show the effects of the calcination temperature on the catalytic activity of W_0_V_5_TiO_2_ catalysts (Figure 10a) and W_5_V_5_TiO_2_ catalysts (Figure 10b). As shown in Figure 10a, the catalytic activity of W_0_V_5_TiO_2_ decreases when calcined above 500 °C. This decline is due to the changes in crystal structure (anatase-to-rutile transformation), morphology, and surface area reduction of the W_0_V_5_TiO_2_ catalysts with an increasing calcination temperature. In contrast, Figure 10b shows that the presence of W on the catalyst may block or inhibit catalyst activity after calcination at 400 °C, since its activity is lower than W_5_V_5_TiO_2_ catalysts calcined at 500 °C and 550 °C. The presence of W on the catalysts at 500 °C, 550 °C, and, to a lesser extent, at 600 °C calcination temperatures, however, preserves the activity of the catalysts for DMS oxidation. Since W inhibits TiO_2_ anatase-to-rutile phase transformation, it preserves the presence of V on anatase TiO_2_ and, thus, catalyst activity at higher calcination temperatures.

**Figure 11:**
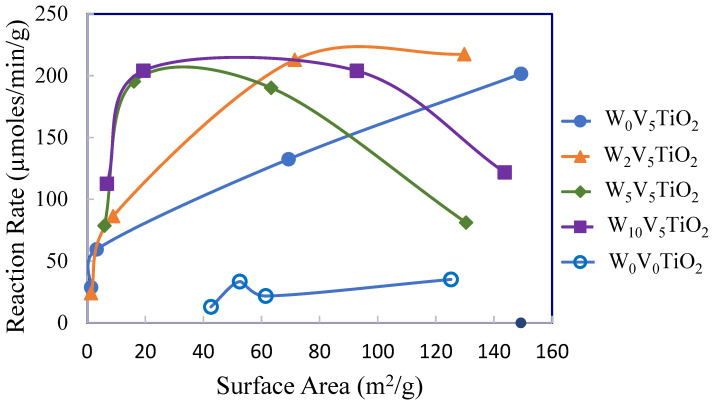
Reaction rate (µmoles/min/g) of DMS at 200 °C as a function of the catalyst composition and surface area.

**Figure 12:**
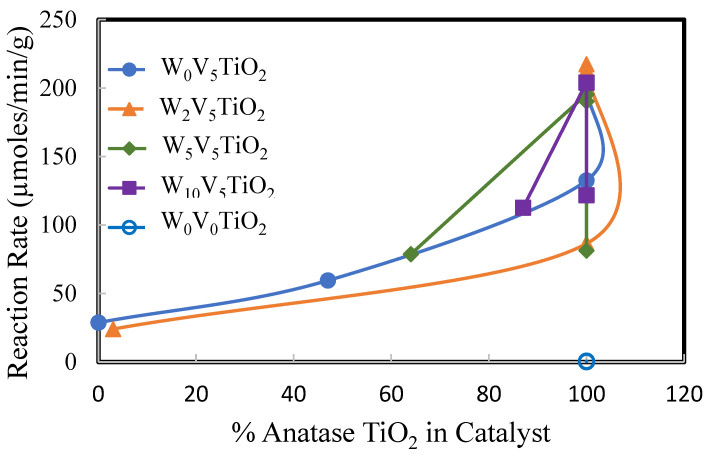
Reaction rate (µmoles/min/g) of DMS at 200 °C as a function of the percent of anatase.

**Figure 13:**
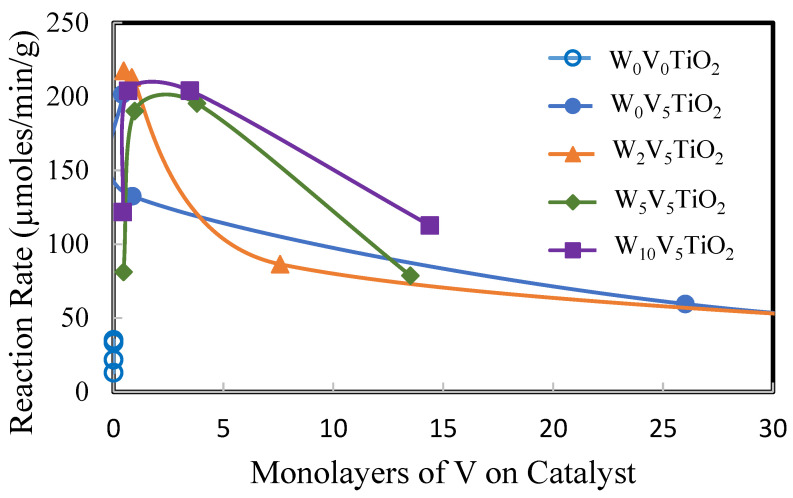
Reaction rate (µmoles/min/g) of DMS oxidation at 200 °C as a function of the monolayers of V on the catalyst.

Figure 11, Figure 12 and Figure 13 show the reaction rate of DMS oxidation at 200 °C as functions of surface area (Figure 11), percentage anatase TiO_2_ (Figure 12), and coverage of catalyst with V as defined by monolayers of V in this manuscript (Figure 13). These figures show that TiO_2_ by itself is not reactive for DMS oxidation at 200 °C. This is evidenced by its relatively low apparent reaction rate compared to the W_x_V_5_TiO_2_ catalysts.

When comparing the reaction rates of the W_0_V_5_TiO_2_ catalyst following calcination at 400 °C and 500 °C in Figure 11, the decrease in surface area from 150 m^2^/g to 75 m^2^/g is likely the reason for the observed decrease in catalytic activity. At these points, the catalyst is still 100% anatase TiO_2_. This demonstrates that the surface area impacts the apparent reaction rate for W_0_V_5_TiO_2_ catalysts.

Comparing the reaction rates of the catalysts following calcination at 400 °C (highest surface area data point for each catalyst), W_0_V_5_TiO_2_ and W_2_V_5_TiO_2_ catalysts are most reactive. These catalysts have strong interactions between vanadia species and the anatase TiO_2_ support. This is supported by Haber et al. (1986) [73], who stated that the structure of vanadium oxygen species determines the oxygen capacity of the vanadia layer, and the reactivity may be significantly related to the transfer of oxygen between the gas phase and catalyst. TiO_2_ has a high concentration of monovanadate species on its surface, which gives it a high overall oxygen capacity. This property would give the catalyst an enhanced redox capacity due to its labile oxygen species.

The WO_3_ species in the W_5_V_5_TiO_2_ and W_10_V_5_TiO_2_ catalysts following calcination at 400 °C are less active than the same catalysts calcined at 500 °C and 550 °C, even though their surface areas decrease considerably with the calcination temperature. A reason for this observation may be that W species are blocking a fraction of the V species interactions with anatase TiO_2_ following calcination at 400 °C. In addition, the higher calcination temperature may enhance the mobility of vanadia species on the surface of the catalysts, allowing for more intimate interactions between vanadia species and anatase TiO_2_ and/or W-V-TiO_2_ interactions, thus creating synergistic benefits.

The highest reaction rates occur on catalysts that are 100% anatase (Figure 11 and Figure 12), even though their surface areas range from 20 m^2^/g to 150 m^2^/g, depending upon the catalyst composition. This gives evidence that the V species over the surface of anatase TiO_2_ significantly contributes to the activity of the catalyst, and its contribution to catalyst activity is greater than that of surface area. While the surface area has a role in apparent reactivity, it is less significant than the interactions of W, V, and anatase TiO_2_.

From Figure 13, the coverage of V on the surface of the catalyst also appears to significantly contribute to the reactivity of the catalyst. The optimal V loading on anatase TiO_2_ is between 0.5 and 4 monolayers. Below 0.5 monolayers of V, activity increases with increasing V. Above four monolayers, the reactivity appears to decrease, likely due to phase transformation and morphological changes. According to Saleh et al. [74], the state of vanadia species on anatase TiO_2_ is strongly dependent upon the calcination temperature and the morphology of the supported vanadia phase. Catalytic activity is enhanced when a complete monolayer of surface vanadia exists on anatase TiO_2_.

## 3. Discussion

This study systematically investigates the morphological, structural, and crystallographic evolution of W_x_V_5_TiO_2_ catalysts as a function of WO_3_ loading and the calcination temperature, ranging from 400 °C to 600 °C. This information is critical for understanding catalytic activity and deactivation mechanisms on this catalyst system.

The anatase-to-rutile transformation is a critical process influencing the thermal, optical, and catalytic properties of TiO_2_. Understanding the theoretical mechanisms underlying this phase transition is essential for tailoring TiO_2_-based materials for various technological applications. The anatase-to-rutile transition is a thermally-activated reconstructive polymorphic phase transition, which involves the breaking and reforming of Ti–O bonds, significant atomic rearrangement, and a change in the unit cell symmetry [75].

Phase transformation of nanocrystalline anatase-to-rutile occurs via combined interface and surface nucleation. The driving force for anatase-to-rutile transition is the reduction in the Gibbs free energy (ΔG) of the system, with rutile being the lower-energy configuration at elevated temperatures [76]. At the atomic scale, the phase transition proceeds through the formation of rutile nuclei within anatase grains, followed by the diffusion and rearrangement of Ti^4+^ and O^2−^ ions, leading to the transformation of the octahedral framework, and finally the growth of rutile domains. This process involves overcoming an activation energy barrier related to the substantial atomic reorganization required for the phase transition [77].

The catalytic activity of these materials is closely associated with V loading on anatase TiO_2_. However, the incorporation of V significantly accelerates morphological changes and facilitates the anatase-to-rutile phase transformation. Specifically, nanoscale (~20 nm) spherical anatase crystals undergo restructuring into larger (>1 μm) rutile structures as the calcination temperature increases from 400 °C to, indicating that V presence on the TiO_2_ surface compromises the thermal stability of the anatase phase.

Conversely, the addition of tungsten oxide (WO_3_) in W_x_V_5_TiO_2_ formulations enhances the thermal stability of the catalyst by inhibiting both morphological alterations and phase transitions across the same temperature range. In addition, Jung et al. [78] found that the introduction of tungsten into V/TiO_2_ catalysts resulted in enhanced reducibility and oxygen mobility through interactions between V and W, which lowered the activation barrier for dichloroethylene oxidation. This suggests that WO_3_ incorporation into the anatase TiO_2_ support not only extends the operational temperature window over which the catalyst retains its structural integrity and activity, but there may be synergistic effects between W, V, and TiO_2_ that enhance oxygen mobility, catalyst reducibility, and hence, catalyst activity.

W_x_V_5_TiO_2_ catalysts with V loading in the range of 0.5 to 4 monolayers of V on anatase TiO_2_ exhibit effective catalytic activity for the oxidation of dimethyl sulfide (DMS). V loadings exceeding seven monolayers promote significant restructuring of the anatase phase, leading to phase transformation into rutile TiO_2_ and, consequently, a loss of the desirable structural properties associated with high catalytic performance.

The activity in this study was assessed using DMS as a probe molecule, but performance under real-world conditions involving multi-component VOC mixtures, humidity, or varying oxygen levels, simulating industrial or environmental exhaust conditions, should be addressed in the future. The use of in situ Raman or XRD under reaction conditions would provide insights into the dynamic behavior of the catalysts during DMS oxidation, particularly phase stability, surface restructuring, and the formation of reaction intermediates.

## 4. Materials and Methods

### 4.1. Materials Used

Ishihara-ST01 TiO_2_ was used as the support material, and it was purchased from Ishihara Sangyo Kaisha Ltd. (Osaka, Japan). The specific surface area of Ishihara ST01 TiO_2_ powder is approximately 300 m^2^/g, and it is 100% anatase, with a primary anatase crystal size of about 7 nm [79]. Ammonium metavanadate was purchased from Fisher Scientific (Waltham, MA, USA), and tungstic acid and ammonium hydroxide were purchased from Sigma-Aldrich (Burlington, MA, USA); all chemicals were used as received.

### 4.2. Synthesis of Catalysts

**Figure 14:**
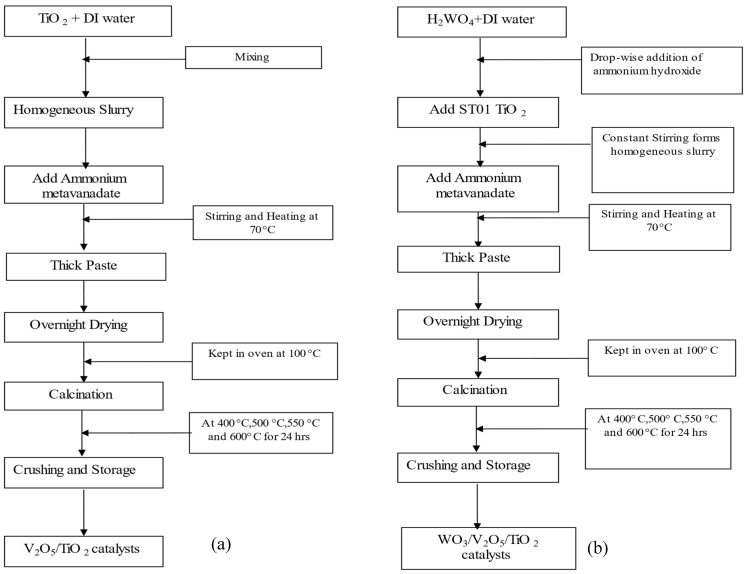
Catalyst synthesis method flow charts. (**a**) W_0_V_5_TiO_2_ catalysts and (**b**) W_x_V_5_TiO_2_ catalysts, where x = 2, 5, and 10 represent W/Ti mass ratios of 0.02, 0.05, and 0.10.

Flow charts to illustrate the methods of catalyst synthesis are provided in Figure 14a,b for W_0_V_5_TiO_2_ and W_x_V_5_TiO_2_ catalysts, respectively.

Briefly, the catalysts were prepared using a wet incipient method, and the amounts of each chemical added are provided in Table S1. For catalysts prepared without the WO_3_ dopant, 5 grams of Ishihara ST01 TiO_2_ was added to 60 mL of deionized water to form an aqueous slurry in a beaker. Constant magnetic stirring was carried out to maintain a uniform slurry. An appropriate amount of ammonium metavanadate was added to obtain a V/Ti mass ratio of 0.05. The slurry was heated to 70 °C under constant stirring until a thick paste was formed. The paste was dried in a pre-heated oven at 100 °C overnight. The dried paste was then crushed to form a fine powder and was divided into four equal parts and calcined in air at 400 °C, 500 °C, 550 °C, and 600 °C for 24 h. The heating rate during calcination was not monitored, but the final calcination temperature was reached within 15 min.

To incorporate the WO_3_ dopant, the catalyst synthesis method was slightly modified. Tungstic acid (H_2_WO_4_) was added to 60 mL of deionized water in a quantity that would obtain W/Ti mass ratios ranging from 0 to 0.10. Ammonium hydroxide was added drop-wise to the solution to ensure the solubility of tungstic acid. After tungstic acid was completely dissolved, a weighed amount of ST01 Ishihara TiO_2_ was added to the solution to form slurry. Ammonium metavanadate was added to the slurry to obtain a V/Ti mass ratio of 0.05. The rest of the procedure as described above was followed. Note that all the W_x_V_5_TiO_2_ catalysts contained V with a V/Ti mass ratio of 0.05.

### 4.3. Catalyst Characterization

Catalyst characterization was conducted by studying its surface area, morphological properties, and crystal and surface structures. All of these properties were useful in the assessment of how WO_3_ affects the morphology, thermal stability, and catalytic activity of W_x_V_5_TiO_2_ catalysts.

#### 4.3.1. Scanning Electron Microscope (SEM)

Scanning electron microscopy (SEM) was used to provide information about the physical and structural details of the catalysts. The morphologies of W_x_V_5_TiO_2_ catalyst particles were studied using a Zeiss Supra 35 VP FEG SEM (Jena, Germany). Catalyst samples for SEM analyses were prepared by dispersing the samples directly on carbon adhesive tapes. The main focus was to analyze the effects of W/Ti mass ratios (Figure S1) and calcination temperatures on the shape, size, surface texture, and arrangement of the particles.

#### 4.3.2. Transmission Electron Microscope (TEM)

Transmission electron microscopy (TEM) was used to provide additional information about the physical and structural details of the catalysts, as shown in Figures S2–S5. TEM micrographs were taken of selected catalysts using a Zeiss 10C TEM (Jena, Germany). The catalysts were dispersed in acetone before being deposited onto carbon-backed TEM copper grids (Ted Pella, Inc., Redding, CA, USA). 

#### 4.3.3. Raman Spectrometry (Raman)

Raman spectra were collected for unused catalysts under ambient conditions using a Renishaw 2000 confocal Raman microprobe (Oxford, UK). Samples were excited with a HeNe (632 nm) laser. This source was focused onto the sample using a 50× (0.85 N.A.) objective, which produced an approximate beam diameter of two micrometers at the sample. Power at the sample did not exceed 6 mW. The same objective was employed to collect the scattered radiation. Spectra were collected at a 4 cm^−1^ resolution and represent the average of 5 individual scans. The integration time for each spectral element was 30 s. Figure S6 depicts the effects of WO_3_ loading on the catalysts that were calcined at 600 °C.

#### 4.3.4. Energy Dispersive X-ray Analysis (EDX)

Simultaneous back-scattered electron diffraction with EDX was used to identify selected morphological structures on selected catalysts (Figures S7–S9).

Energy dispersive analysis was carried out to investigate the effects of the W/Ti mass ratios and calcination temperature on the final composition of the catalysts. EDX spectra were collected by using an EDAX Genesis 2000. The sample preparation was same as that for SEM. The entire spectra were collected at an accelerating voltage of 20 keV. Elemental analyses are depicted in Figures S10–S12; elemental mapping is provided in Figures S13–S16.

#### 4.3.5. BET Surface Area

A Beckman Coulter SA3100 (Brea, CA, USA) was used to measure the surface area of the catalysts with N_2_ adsorption at 77 K. The samples were outgassed at 120 °C for 45 min prior to the surface area analyses. A second set of BET surface area measurements were conducted on all catalysts using a Micromeritics TriStar II surface area analyzer (Norcross, GA, USA), also with N2 adsorption at 77 K. Adsorption isotherms from this instrument are provided in Figures S17–S21, and the pore sizes, as determined using the BJH model and the desorption data, are provided in Figure S22.

#### 4.3.6. X-ray Diffraction

The diffraction patterns of the catalysts samples in this study were recorded using a Scintag X-ray powder diffractometer (Cupertino, CA, USA) with a Cu Kα radiation source. Measurements were carried out in 2θ range extending from 20° to 60° at the step size of 0.04 and preset time of 0.5 s. XRD was operated at a voltage of 40 kV and filament current of 35 mA. The equipment was attached to a computer installed with software that was used to record and analyze the diffraction data. The diffraction data were plotted (instrument response vs. 2θ) to obtain the spectra. For anatase TiO_2_, the predominant peak (101) was at 2θ = 25.3°, and for rutile TiO_2_, the predominant peak (110) was at 2θ = 27.4°.

Equation (3) was used to calculate the fraction anatase phase of the TiO_2_ using XRD diffractograms, as well as the intensity of the anatase (101) TiO_2_ (Ia) and rutile (110) TiO_2_ (Ir) peaks, as described above. The Debye–Scherrer equation (Equation (4)) was used to calculate the anatase (101) crystal size from the XRD diffractogram, also described above.

#### 4.3.7. Thermal Gravimetric Analyzer (TGA)

TGA analyses were conducted on all catalysts using a TA Instruments Q500 TGA (New Castle, DE, USA). The temperature was ramped at 10 °C/min from ambient temperature to 900 °C under a nitrogen gas atmosphere. The TGA data for catalysts that were calcined at 400 °C are provided in Figure S23, and mass losses as a function of temperature range are summarized in Table S2.

### 4.4. Catalyst Performance

**Figure 15:**
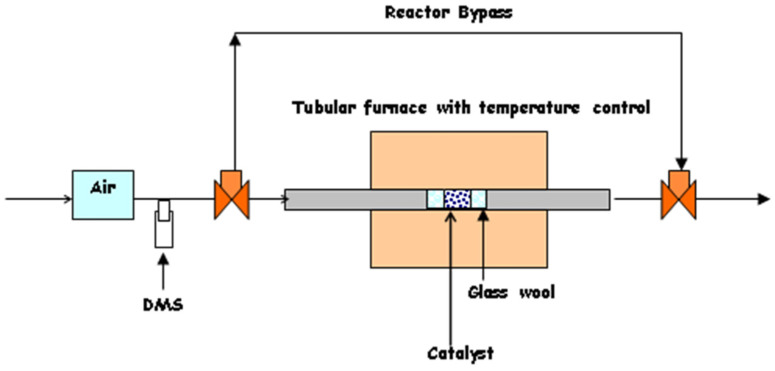
Schematic of the test system used for catalyst performance analysis.

The test system used in this study, depicted in Figure 15, consists of a stainless-steel tubular reactor (ID = 0.32 cm), a diffusion cell to generate dimethyl sulfide vapors, a mass flow controller (MKS Instruments, Andover, MA, USA, model no. M100B12CS1BV) to control the flow rate of air, and a Lindberg/Blue Mini-Mite tube furnace (Thermo Fisher Scientific, Waltham, MA, USA) to control the reaction temperature.

For each experimental trial, the performance of the catalyst was investigated using 50 mg of catalyst that was secured with quartz wool in the middle of a 0.32 cm ID stainless-steel reaction tube. Air, flowing at 20 cubic centimeters per minute (ccpm), was mixed with dimethyl sulfide vapor from the diffusion cell, resulting in a dimethyl sulfide concentration of 16,200 ppm (1.62 vol%). This gas stream was directed through the tubular reactor with a gas hourly space velocity (GHSV) = 3760/h, and the temperature in the reactor varied from 100 °C to 250 °C. The flow of air was measured before and after each experimental trial using a bubble meter. Online sampling valves on the gas chromatograph (GC), with a 1 mL sample loop, was used to analyze the influent and effluent from the reactor. The GC that was used is an Agilent 6890N gas chromatograph (GC) (Santa Clara, CA, USA) equipped with a thermal conductivity detector. A J&W Scientific DB-Wax column (Agilent Santa Clara, CA, USA) [80] was used to separate the components in the reactor effluent. ChemStation software (Revision A.09.01 2001) was used to obtain peak areas. The first readings were taken in triplicate at 100 °C, and subsequent samples were collected in at least duplicates two hours after the reaction temperature was reached.

## Figures and Tables

**Table 1:** Overall summary of the morphological properties of the catalysts.

Catalyst	Catalyst Composition ^1^		Calcination Temperature (°C)	XRD Results		BET Area (m^2^/g)	Pore Size ^4^ (nm)	Monolayers ^5^ of V on TiO_2_
	V/Ti Mass Ratio	W/Ti Mass Ratio		Percent Anatase ^2^ (%)	Anatase Crystal Size ^3^ (nm)			
W_0_V_0_TiO_2_	0	0	400	100	26.5	125 ± 8.2	17.4	0
			500	100	40.1	61.4 ± 13.5	23.2	0
			550	100	42.0	52.5 ± 12	25.3	0
			600	100	45.5	42.6 ± 7.6	26.2	0
W_0_V_5_TiO_2_	0.05	0	400	100	24.5	149 ± 10.9	14.7	0.40 ± 0.03
			500	100	36.7	69.3 ± 0.4	25.6	0.85 ± 0.01
			550	47	112	3.2 ± 2.5	51.2	26.0 ± 20.1
			600	0	0.0	1.3 ± 1	38.6	60.6 ± 43.9
W_2_V_5_TiO_2_	0.05	0.02	400	100	24.2	130 ± 22.3	14.9	0.46 ± 0.08
			500	100	38.0	71.5 ± 0.8	25.8	0.83 ± 0.01
			550	100	91.4	8.9 ± 4.4	49.6	7.58 ± 3.75
			600	3	113	1.3 ± 1.0	43.6	62.4 ± 46.5
W_5_V_5_/TiO_2_	0.05	0.05	400	100	23.0	130 ± 4.8	16.2	0.45 ± 0.02
			500	100	41.9	63.3 ± 15.1	25.3	0.96 ± 0.23
			550	100	69.3	16.1 ± 4.1	39.8	3.79 ± 0.97
			600	64	109	5.95 ± 4.3	37.3	13.5 ± 9.82
W_10_V_5_/TiO_2_	0.05	0.10	400	100	20.7	144 ± 3.8	14.8	0.41 ± 0.01
			500	100	30.7	92.8 ± 25.7	22.2	0.66 ± 0.18
			550	100	61.1	19.3 ± 9.4	47.4	3.47 ± 1.69
			600	87	110	6.76 ± 6.0	42.2	14.4 ± 12.8

^1^ The masses of precursors used for each catalyst is provided in Table S1. ^2^ The percent anatase TiO_2_ is based on Equation (3), as reported in [53]. ^3^ The anatase crystal size is based on the Scherrer equation, Equation (4). ^4^ The pore size is based on the Barrett–Joyner–Halenda (BJH) model and nitrogen desorption data, acquired during BET surface area analyses. ^5^ Monolayers of vanadia species on the surface of the catalyst, as defined by Equations (1) and (2) and [18].

## Data Availability

All raw data supporting this study are available upon request from the corresponding author.

## Supplementary Materials

The following supporting information can be downloaded at: https://www.mdpi.com/article/10.3390/molecules30112436/s1, Table S1. Catalyst synthesis. Table S2. Summary of mass loss as a function of temperature range. Figure S1. SEM images (30,000 magnification) of W_x_V_5_TiO_2_ catalysts calcined in air at 600 °C C for 24 h. (a) Pure TiO_2_; (b) W_0_V_5_TiO_2_; (c) W_2_V_5_TiO_2_; (d) W_5_V_5_TiO_2_; (e) W_10_V_5_TiO_2_. Figure S2. TEM image of W_0_V_5_TiO_2_ calcined at 400 °C. Figure S3. TEM image of W_0_V_5_TiO_2_ calcined at 500 °C. Figure S4. TEM image of W_5_V_5_TiO_2_ calcined at 400 °C. Figure S5. TEM image of W_5_V_5_TiO_2_ calcined at 500 °C. Figure S6. Raman spectra of W_x_V_5_TiO_2_ catalysts calcined at 600 °C. (a) 100 cm^−1^ to 1400 cm^−1^; (b) enlarged area for selected catalysts from 700 cm^−1^ to 1050 cm^−1^. Figure S7. Simultaneous Electron Backscatter Diffraction (EBSD) with EDS on W_5_V_5_TiO_2_. Figure S8. Simultaneous Electron Backscatter Diffraction (EBSD) with EDS on W_0_V_5_TiO_2_. Figure S9. Simultaneous Electron Backscatter Diffraction (EBSD) with EDS on W_5_V_5_TiO_2_. Figure S10. EDS profile for TiO_2_ calcined at 600 °C. Figure S11. EDS profile for V_5_TiO_2_ calcined at 600 °C. Figure S12. EDS profile for W_5_V_5_TiO_2_ calcined at 600 °C. Figure S13. Elemental (a) mapping and (b) quantification of W_0_V_5_TiO_2_ calcined at 500 °C using EDX. Working distance = 10 mm; Aperture = 60 μm; EHT (accelerating voltage) = 15 kV. Figure S14. Elemental (a) mapping and (b) quantification of W_0_V_5_TiO_2_ calcined at 600 °C using EDX. Working distance = 10 mm; Aperture = 60 μm; EHT (accelerating voltage) = 15 kV. Figure S15. Elemental (a) mapping and (b) quantification of W_5_V_5_TiO_2_ calcined at 500 °C using EDX. Working distance = 10 mm; Aperture = 60 μm; EHT (accelerating voltage) = 15 kV. Figure S16. Elemental (a) mapping and (b) quantification of W_5_V_5_TiO_2_ calcined at 600 °C using EDX. Working distance = 10 mm; Aperture = 60 μm; EHT (accelerating voltage) = 15 kV. Figure S17. Nitrogen adsorption isotherms for TiO_2_. Figure S18. Nitrogen adsorption isotherms for W_0_V_5_TiO_2_. Figure S19. Nitrogen adsorption isotherms for W_2_V_5_TiO_2_. Figure S20. Nitrogen adsorption isotherms for W_5_V_5_TiO_2_. Figure S21. Nitrogen adsorption isotherms for W_10_V_5_TiO_2_. Figure S22. Pore size as a function of calcination temperature and catalyst composition. Pore size determined by nitrogen desorption data and the Barrett-Joyner-Halenda (BJH) model. Figure S23. TGA analyses of catalysts that had been calcined in air at 400 °C for 24 h. (a) Mass fraction remaining vs temperature; (b) derivative d(mass)/d(temp) (g/°C).

## Abbreviations

The following abbreviations are used in this manuscript:

BET	Brunauer–Emmett–Teller surface area analysis method
DMS	Dimethyl sulfide
EDX	Energy dispersion X-ray spectroscopy
FTIR	Fourier transform infrared spectroscopy
SEM	Scanning electron microscope
VOCs	Volatile organic compounds
W_x_V_5_TiO_2_	WO_3_/V_2_O_5_/TiO_2_ catalysts with x = mass ratio W/Ti; 5 = mass ratio V/Ti
XRD	X-ray diffraction
