# Supplementary material for: An Investigation of WO3/V2O5/TiO2 Catalysts: Effects of WO3 on Morphology, Thermal Stability, and Activity for the Catalytic Oxidation of Dimethyl Sulfide
Source: Molecules. 2025 Jun 2;30(11):2436. doi: 10.3390/molecules30112436 (PMC12155995; doi:10.3390/molecules30112436)
Supplement: Supplementary file 1 [file molecules-30-02436-s001.zip › molecules-3636227-supplementary.pdf]

Supplementary Information

Table S1. Catalyst synthesis

Table S2. Summary of mass loss as a function of temperature range.

Figure S1. SEM images (30,000 magnification) of $W_xV_5TiO_2$ catalysts calcined in air at 600°C C for 24 hours. (a) Pure TiO_2; (b) $W_0V_5TiO_2$; (c) $W_2V_5TiO_2$; (d) $W_5V_5TiO_2$; (e) $W_{10}V_5TiO_2$

Figure S2. TEM image of $W_0V_5TiO_2$ calcined at 400°C

Figure S3. TEM image of $W_0V_5TiO_2$ calcined at 500°C

Figure S4. TEM image of $W_5V_5TiO_2$ calcined at 400°C

Figure S5. TEM image of $W_5V_5TiO_2$ calcined at 500°C

Figure S6. Raman spectra of $W_xV_5TiO_2$ catalysts calcined at 600°C. (a) 100 cm^{-1} to 1400 cm^{-1}; (b) enlarged area for selected catalysts from 700 cm^{-1} to 1050 cm^{-1}.

Figure S7. Simultaneous Electron Backscatter Diffraction (EBSD) with EDS on $W_5V_5TiO_2$

Figure S8. Simultaneous Electron Backscatter Diffraction (EBSD) with EDS on $W_0V_5TiO_2$.

Figure S9. Simultaneous Electron Backscatter Diffraction (EBSD) with EDS on $W_5V_5TiO_2$.

Figure S10. EDS profile for TiO_2 calcined at 600°C.

Figure S11. EDS profile for V_5TiO_2 calcined at 600°C.

Figure S12. EDS profile for $W_5V_5TiO_2$ calcined at 600°C.

Figure S13. Elemental (a) mapping and (b) quantification of $W_0V_5TiO_2$ calcined at 500°C using EDX. Working distance = 10 mm; Aperture = 60µm ; EHT (accelerating voltage) = 15 kV.

Figure S14. Elemental (a) mapping and (b) quantification of $W_0V_5TiO_2$ calcined at 600°C using EDX. Working distance = 10 mm; Aperture = 60µm ; EHT (accelerating voltage) = 15 kV.

Figure S15. Elemental (a) mapping and (b) quantification of $W_5V_5TiO_2$ calcined at 500°C using EDX. Working distance = 10 mm; Aperture = 60µm ; EHT (accelerating voltage) = 15 kV.

Figure S16. Elemental (a) mapping and (b) quantification of $W_5V_5TiO_2$ calcined at 600°C using EDX. Working distance = 10 mm; Aperture = 60µm ; EHT (accelerating voltage) = 15 kV.

Figure S17. Nitrogen adsorption isotherms for TiO_2

Figure S18. Nitrogen adsorption isotherms for $W_0V_5TiO_2$

Figure S19. Nitrogen adsorption isotherms for $W_2V_5TiO_2$

Figure S20. Nitrogen adsorption isotherms for $W_5V_5TiO_2$

Figure S21. Nitrogen adsorption isotherms for $W_{10}V_5TiO_2$

Figure S22. Pore size as a function of calcination temperature and catalyst composition. Pore size determined by nitrogen desorption data and the Barrett-Joyner-Halenda (BJH) model.

Figure S23. TGA analyses of catalysts that had been calcined in air at 400°C for 24 hours. (a) Mass fraction remaining vs temperature; (b) derivative d(mass)/d(temp) (g/°C)

Catalyst Synthesis

The catalysts were prepared by wet incipient methods. 5 g of TiO_2 were mixed with 60 mL deionized water in a beaker. The beaker was set on a stirring hot plate and continuously mixed under heat (70°C). Measured masses of ammonium metavanadate and tungstic acid were added to the slurry while mixing. A few drops of ammonium hydroxide were also added to help solubilized the tungstic acid. The slurry was mixed and heated until enough water evaporated to make a paste. The paste was dried in an oven at 100°C overnight. The dried powders were crushed and separated into 1 g aliquots. The 1 g samples of each catalyst were calcined for 24 hours at 400°C, 500°C, 550°C, or 600°C.

Table S1. Catalyst synthesis

Catalyst ID	Ishihara ST-01 Anatase TiO_2	Deionized Water	Ammonium Metavanadate	Tungstic Acid	Ammonium Hydroxide
$W_0V_0TiO_2$	5 g	60 mL	0 g	0 g	0 drops
$W_0V_5TiO_2$	5 g	60 mL	0.575 g	0 g	0 drops
$W_2V_5TiO_2$	5 g	60 mL	0.575 g	0.136 g	<5 drops
$W_5V_5TiO_2$	5 g	60 mL	0.575 g	0.340 g	<5 drops
$W_{10}V_5TiO_2$	5 g	60 mL	0.575 g	0.680 g	<5 drops

SEM micrograph to further show the effects of W on V_5TiO_2 catalysts

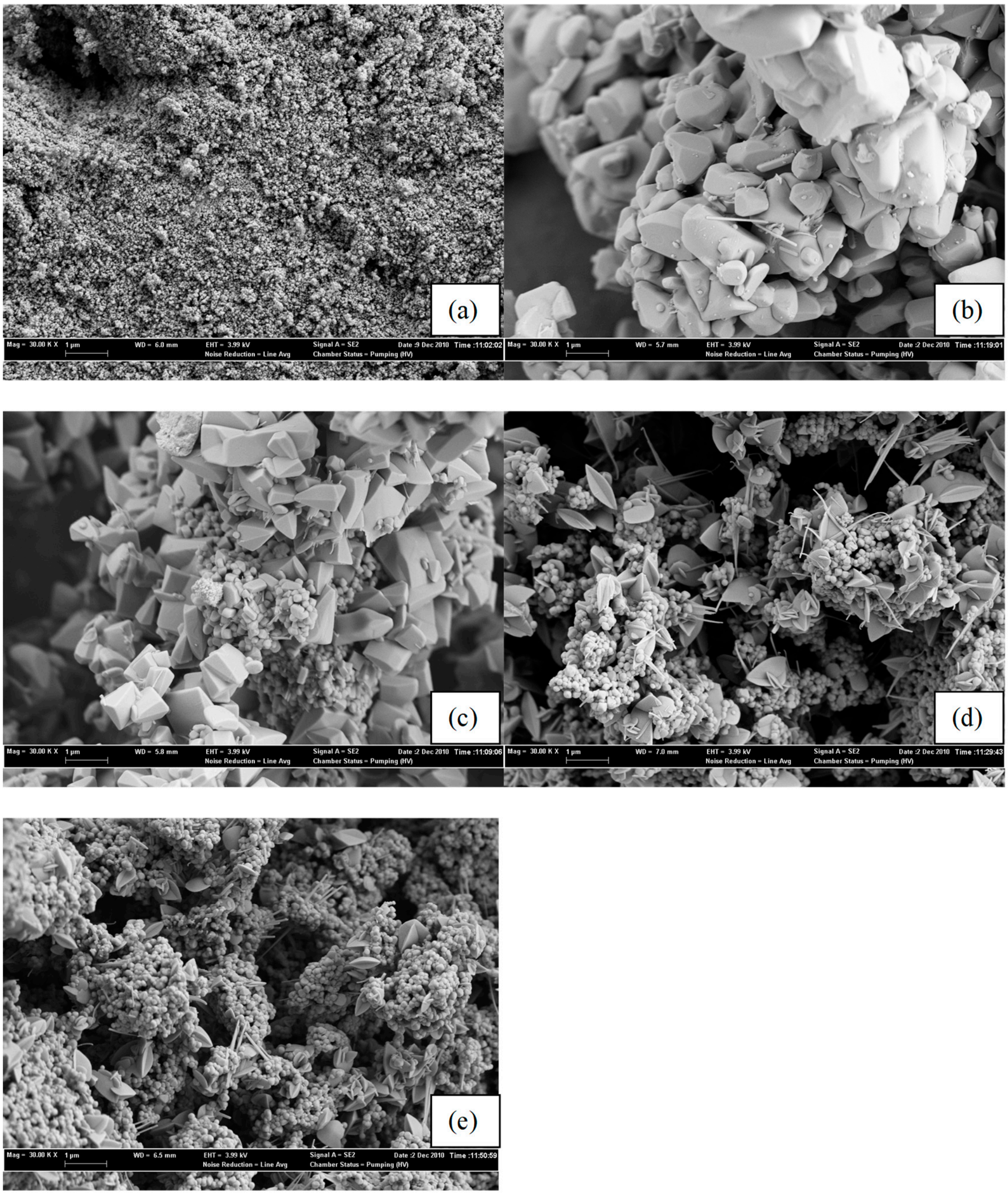

Figure S1. SEM images (30,000 magnification) of $W_xV_5TiO_2$ catalysts calcined in air at 600°C C for 24 hours. (a) Pure TiO_2; (b) $W_0V_5TiO_2$; (c) $W_2V_5TiO_2$; (d) $W_5V_5TiO_2$; (e) $W_{10}V_5TiO_2$

Figure S1. clearly shows that the addition of WO_3 inhibits phase transformation in anatase TiO_2. In this figure, the spherical features are anatase TiO_2 (see Figure S2.) whereas the larger plate-like and block-like features are rutile TiO_2 (see Figure S3.).

Transmission Electron Microscopy (TEM) on Selected Samples

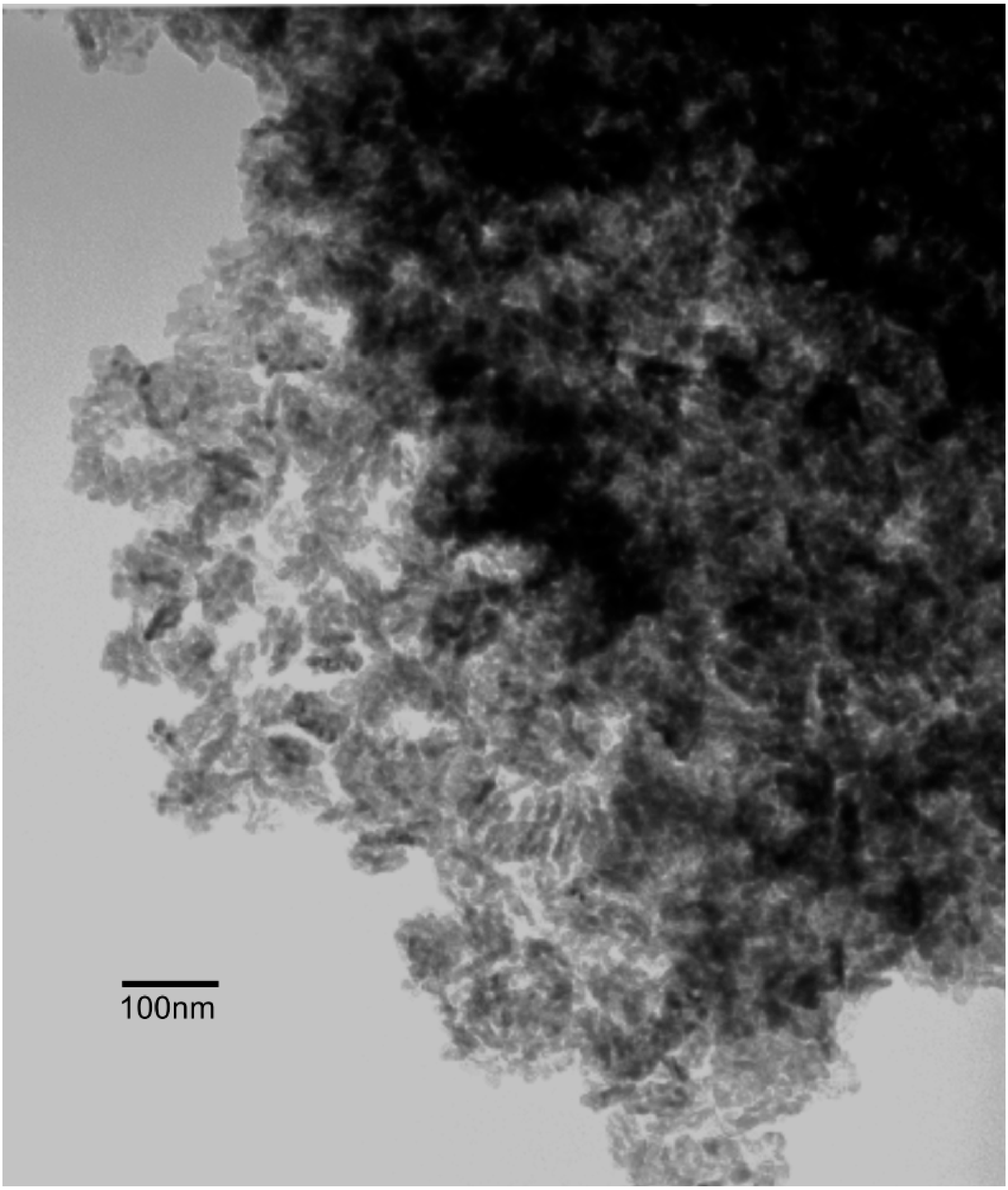

Figure S2. TEM image of $W_0V_5TiO_2$ calcined at 400°C

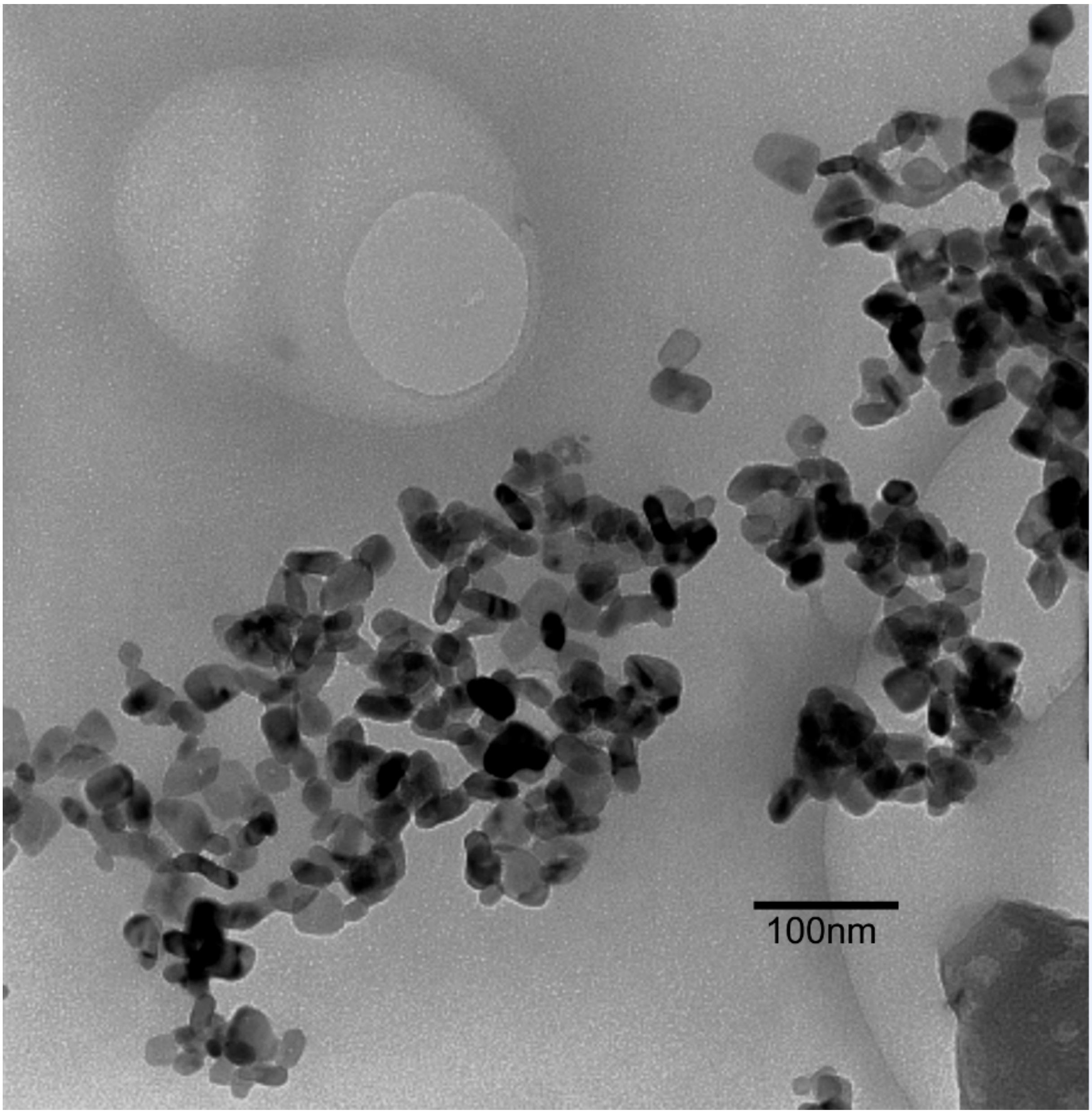

Figure S3. TEM image of $W_0V_5TiO_2$ calcined at 500°C

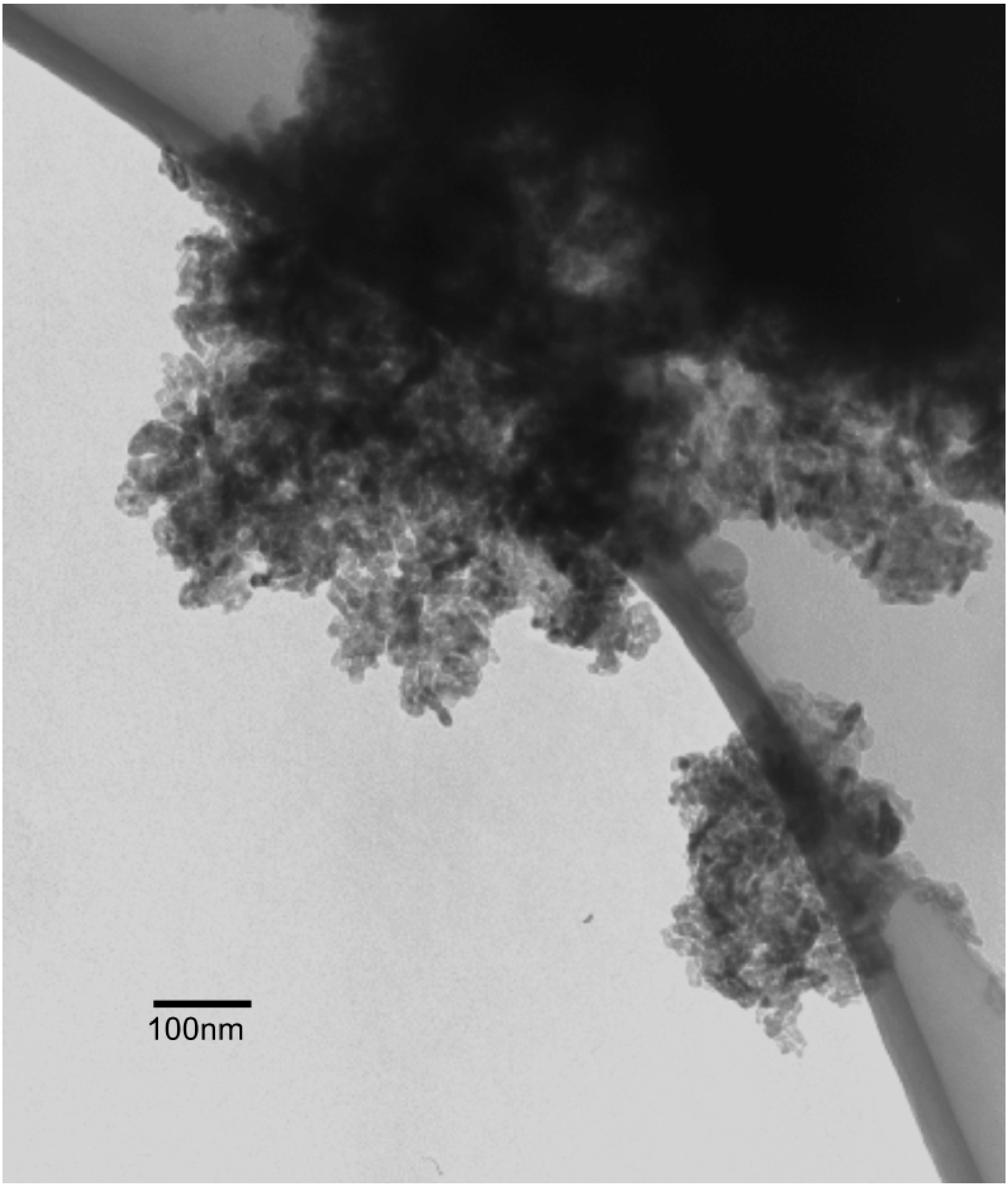

Figure S4. TEM image of $W_5V_5TiO_2$ calcined at 400°C

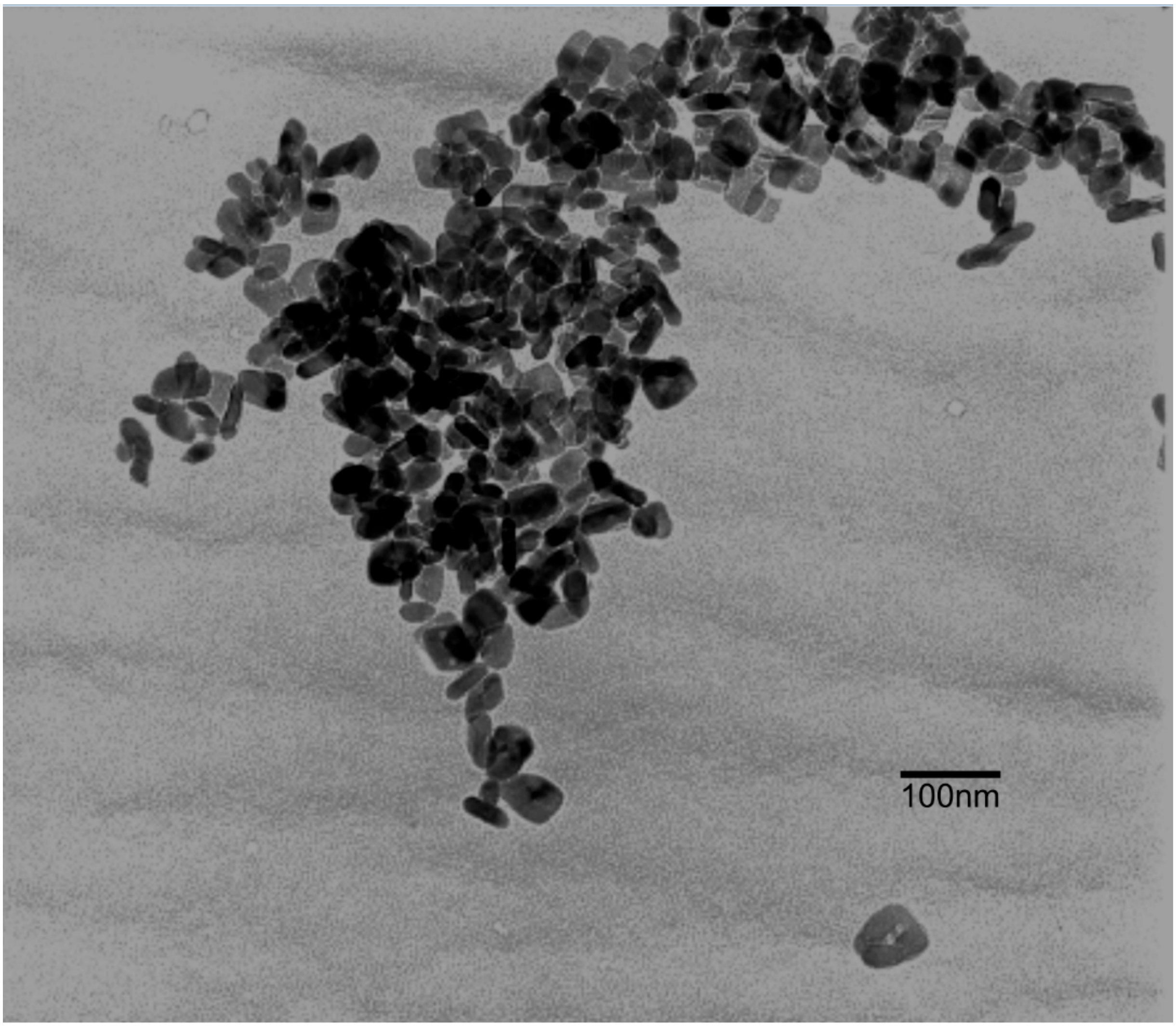

Figure S5. TEM image of $W_5V_5TiO_2$ calcined at 500°C

Raman Spectroscopy

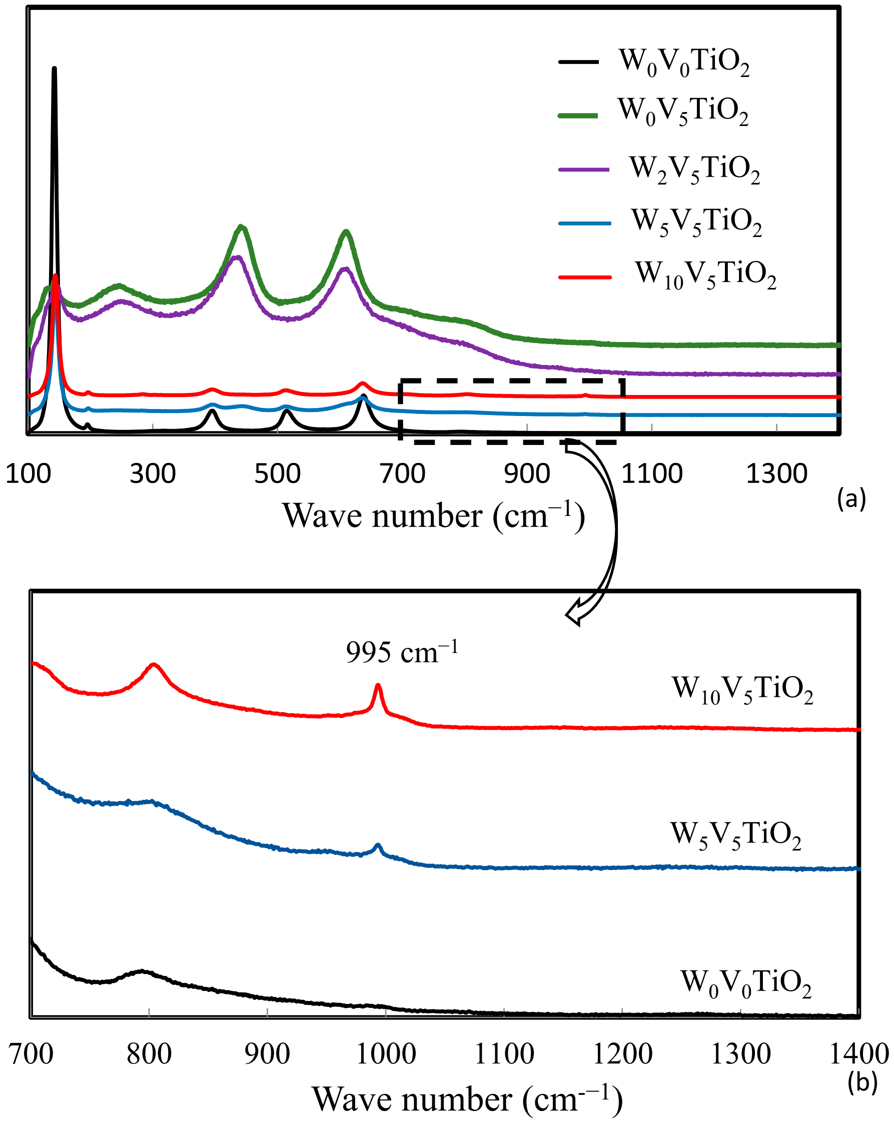

Figure S6. Raman spectra of $W_xV_5TiO_2$ catalysts calcined at 600°C. (a) 100 cm^{-1} to 1400 cm^{-1}; (b) enlarged area for selected catalysts from 700 cm^{-1} to 1050 cm^{-1}.

Energy dispersion spectroscopy (EDS) for selected catalysts

PhaseID Report

User:
Sample:

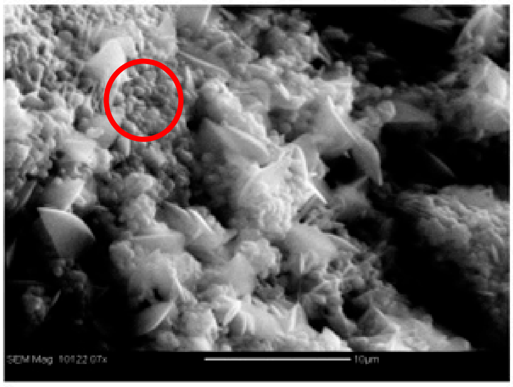

Composition:

Definitely present:
O, Ti

Maybe present:
V, W

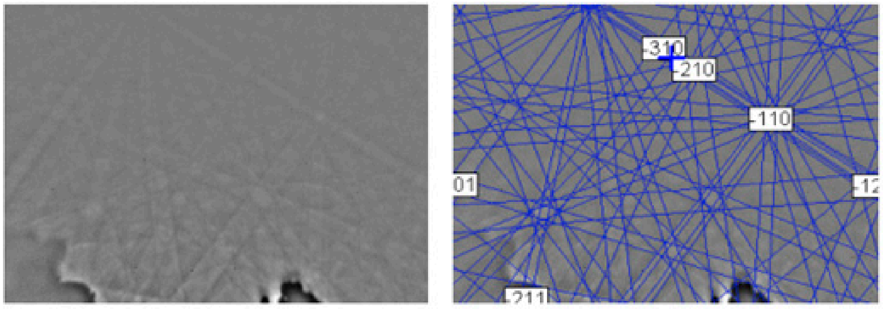

BC = 174, BS = 67

MAD = 0.890°, Orientation = (145.4, 69.8, 82.6)°, Spec. plane ~ (-61-1)

Phase:

Name:	Anatase, syn
Data source:	J. Am. Chem. Soc. [JACSAT], (1955), vol. 77, page 4708
Database name:	PDF2 database
Laue and space group no.:	5, 4/mmm; 136
Unit cell lengths:	3.79, 3.79, 9.51 Å
Unit cell angles:	90.0, 90.0, 90.0 °
Composition:	O(66.7 at%), Ti(33.3)

Figure S7. Simultaneous Electron Backscatter Diffraction (EBSD) with EDS on $W_5V_5TiO_2$.

PhaseID Report

User:
Sample:

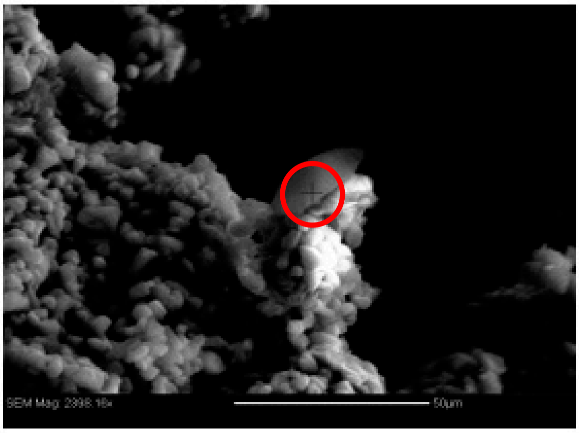

Composition:

Definitely present:
O, Ti

Maybe present:
V, W

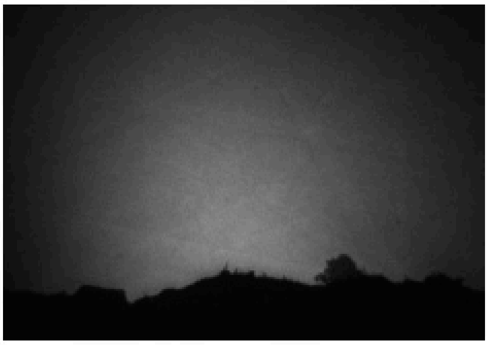

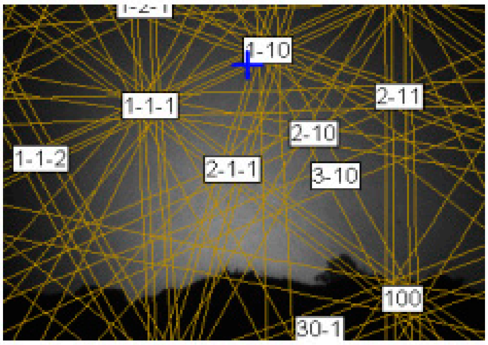

BC = 247, BS = 36

MAD = 0.557°, Orientation = (118.6, 82.6, 65.5)°, Spec. plane ~ (2-41),

Phase:

Name:	Titanium Vanadium Oxide
Data source:	Tex. J. Sci. [TJSCAU], (1951), vol. 1, page 82
Database name:	PDF2 database
Laue and space group no.:	5, 4/mmm; 136
Unit cell lengths:	4.58, 4.58, 2.95 Å
Unit cell angles:	90.0, 90.0, 90.0 °
Composition:	O(66.7 at%), Ti(16.7), V(16.7)

Figure S8. Simultaneous Electron Backscatter Diffraction (EBSD) with EDS on $W_0V_5TiO_2$.

PhaseID Report

User:
Sample:

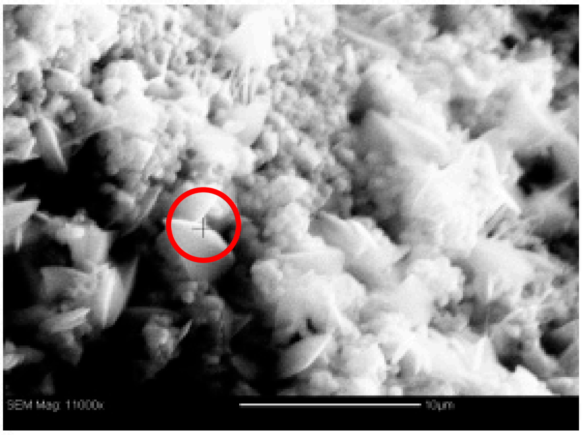

Composition:

Definitely present:
O, Ti

Maybe present:
V, W

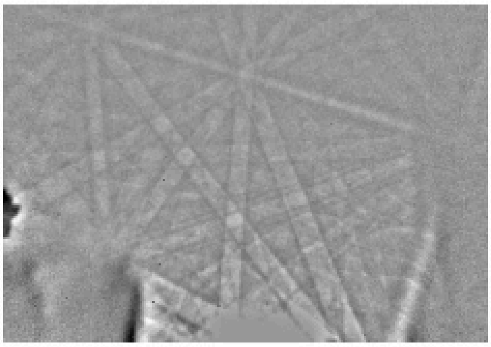

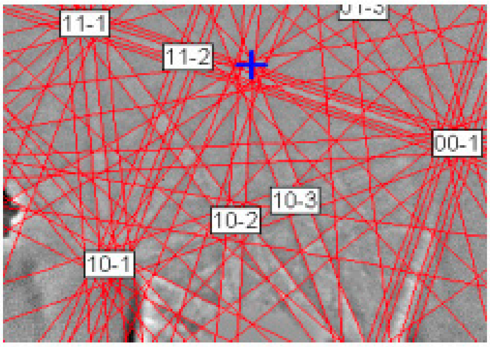

BC = 240, BS = 85

MAD = 0.505°, Orientation = (46.7, 49.3, 80.3)°, Spec. plane ~ (15-7)

Phase:

Name:	Rutile
Data source:	[Rutile.cry]
Database name:	HKL phases
Laue group:	5, 4/mmm
Unit cell lengths:	4.59, 4.59, 2.96 Å
Unit cell angles:	90.0, 90.0, 90.0 °
Composition:	O(66.7 at%), Ti(33.3)

Figure S9. Simultaneous Electron Backscatter Diffraction (EBSD) with EDS on $W_5V_5TiO_2$.

EDS analyses were conducted on selected samples, as shown below. The mapping of elements showed that the vanadium and tungsten were well dispersed over the catalyst surface. The elemental quantification supports the desired catalyst composition.

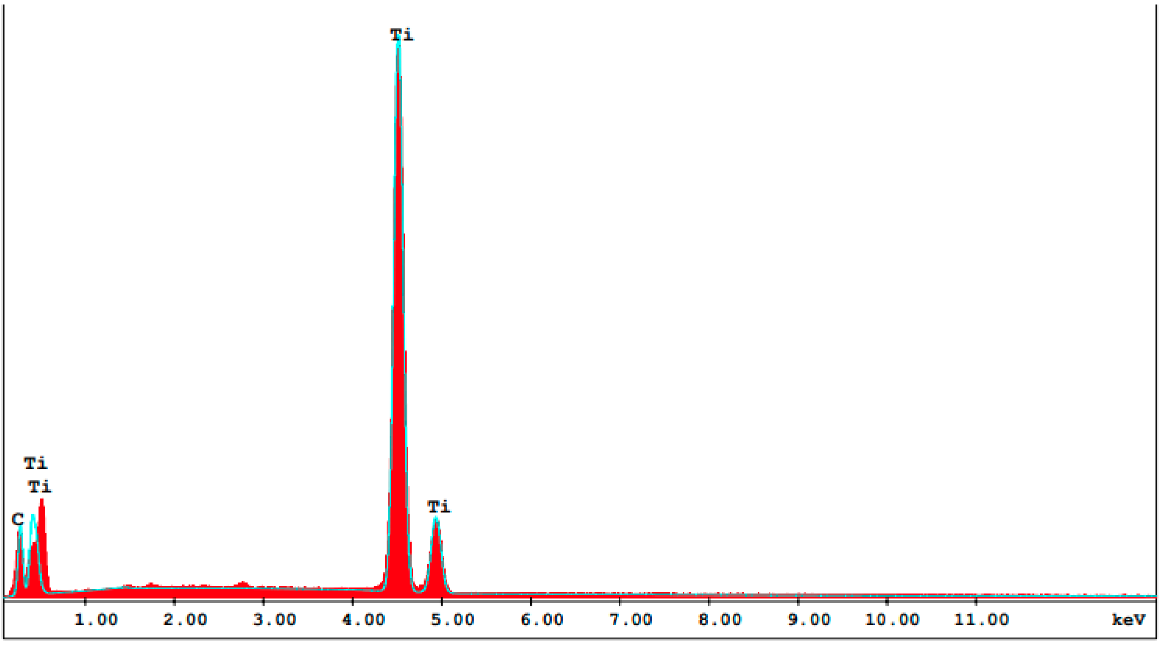

EDAX ZAF Quantification (Standardless)
Oxides
SEC Table : Default

Element	Wt %	Mol %	K-Ratio	Z	A	F
TiO2	100.00	100.00	0.5709	0.9345	1.0190	1.0000
Total	100.00	100.00				

Element	Net Inte.	Bkgd Inte.	Inte. Error	P/B
TiK	694.66	8.65	0.27	80.26

Figure S10. EDS profile for TiO_2 calcined at 600°C.

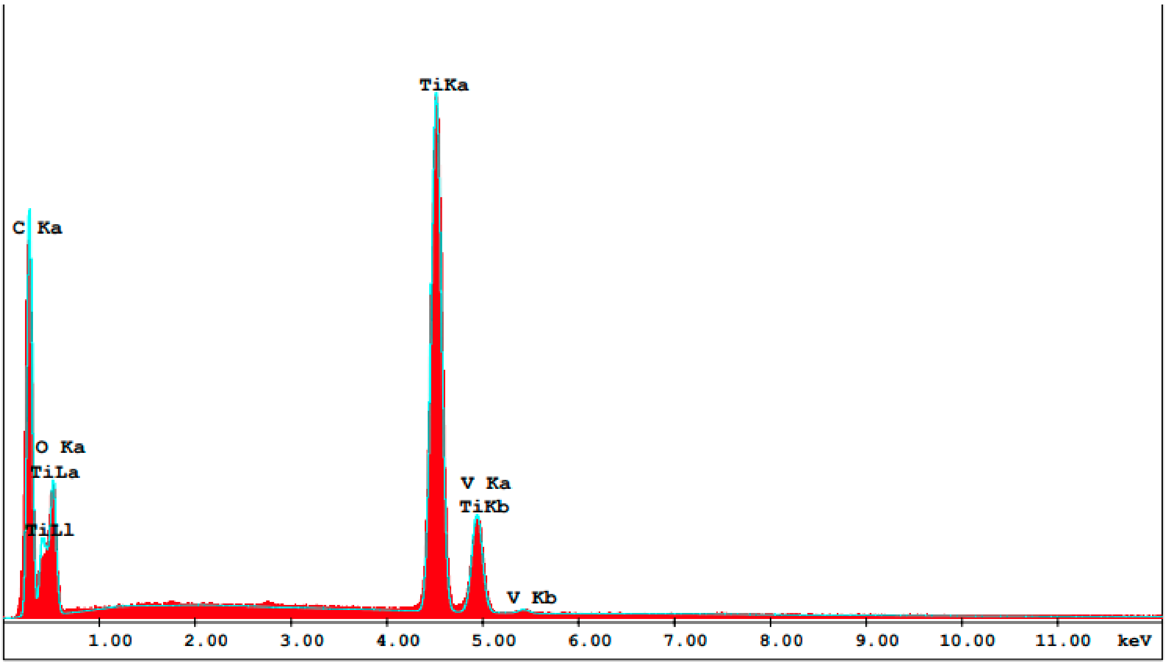

EDAX ZAF Quantification (Standardless)
Oxides
SEC Table : Default

Element	Wt %	Mol %	K-Ratio	Z	A	F
TiO2	94.01	97.28	0.5366	0.9347	1.0186	1.0000
V2O5	5.99	2.72	0.0316	0.9174	1.0279	1.0000
Total	100.00	100.00				

Element	Net Inte.	Bkgd Inte.	Inte. Error	P/B
TiK	439.20	7.26	0.33	60.52
V K	23.72	6.17	1.74	3.84

Figure S11. EDS profile for V_5TiO_2 calcined at 600°C.

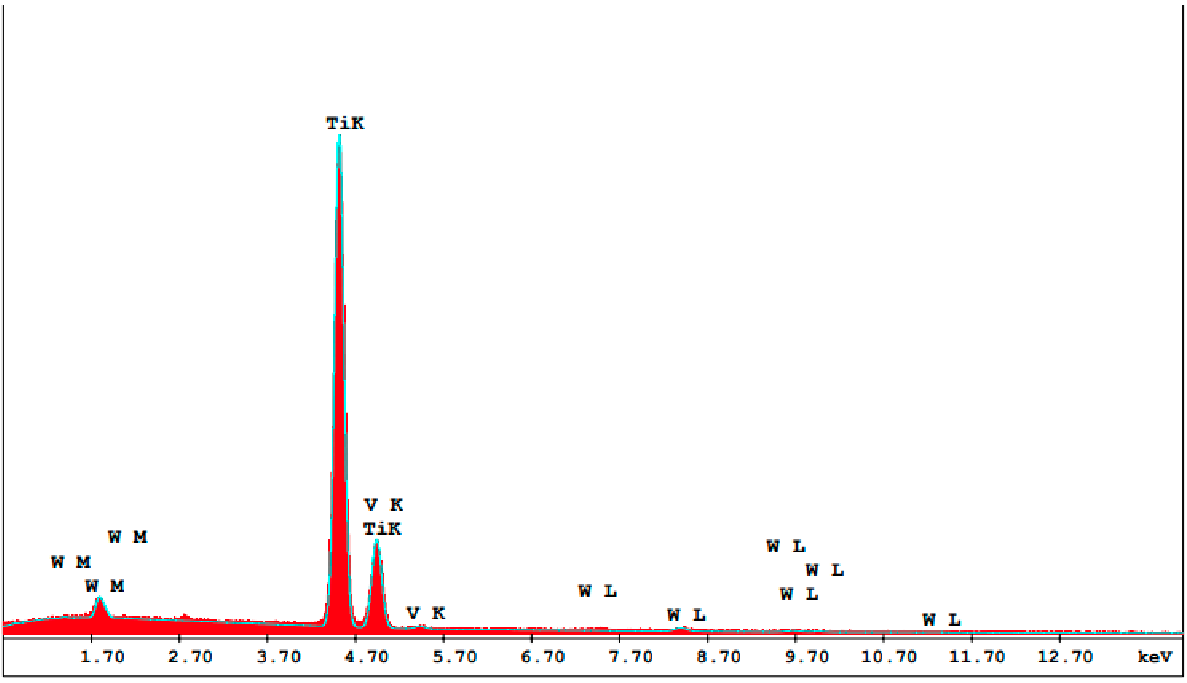

EDAX ZAF Quantification (Standardless)
Oxides
SEC Table : Default

Element	Wt %	Mol %	K-Ratio	Z	A	F
TiO2	92.00	97.07	0.5182	0.9383	1.0002	1.0010
V2O5	5.02	2.33	0.0263	0.9207	1.0131	1.0015
W2O3	2.98	0.60	0.0212	0.7802	1.0294	1.0000
Total	100.00	100.00				

Element	Net Inte.	Bkgd Inte.	Inte. Error	P/B
TiK	1735.97	46.04	0.17	37.71
V K	80.59	40.90	1.12	1.97
W L	13.07	27.22	4.45	0.48

Figure S12. EDS profile for $W_5V_5TiO_2$ calcined at 600°C.

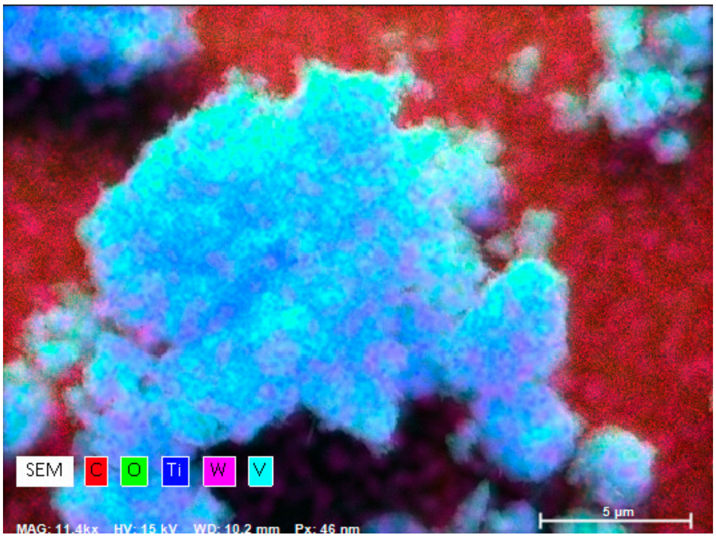

(a)

Map

Element	Line series	Net counts	Mass Concentration /%	Norm. mass concentration /%	Norm. atomic concentration /%	1σ uncertainty /mass%	1σ rel. uncertainty /%
Carbon	K	977494	71.19	57.21	72.54	1.14	1.60
Oxygen	K	137019	27.25	21.90	20.84	0.56	2.04
Titanium	K	488665	24.65	19.80	6.30	0.41	1.65
Tungsten	L	63	0.03	0.02	0.00	0.04	134.14
Vanadium	K	22480	1.33	1.07	0.32	0.03	2.57
		**Sum**	**124.44**	**100.00**	**100.00**		

(b)

Figure S13. Elemental (a) mapping and (b) quantification of $W_0V_5TiO_2$ calcined at 500°C using EDX. Working distance = 10 mm; Aperture = 60µm ; EHT (accelerating voltage) = 15 kV.

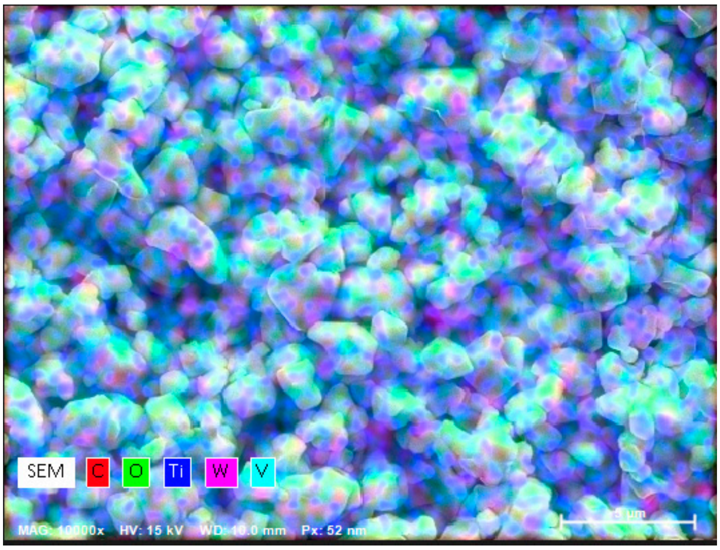

(a)

Map							
Element	Line series	Net counts	Mass Concentration /%	Norm. mass concentration /%	Norm. atomic concentration /%	1σ uncertainty /mass%	1σ rel. uncertainty /%
Carbon	K	2837	0.63	0.72	1.61	0.03	4.49
Oxygen	K	74176	33.41	38.31	64.30	0.71	2.13
Titanium	K	503397	50.67	58.10	32.59	0.89	1.75
Tungsten	L	17	0.02	0.02	0.00	0.07	429.12
Vanadium	K	20486	2.49	2.86	1.51	0.07	2.74
		**Sum**	**87.22**	**100.00**	**100.00**		

(b)

Figure S14. Elemental (a) mapping and (b) quantification of $W_0V_5TiO_2$ calcined at 600°C using EDX. Working distance = 10 mm; Aperture = 60μm ; EHT (accelerating voltage) = 15 kV.

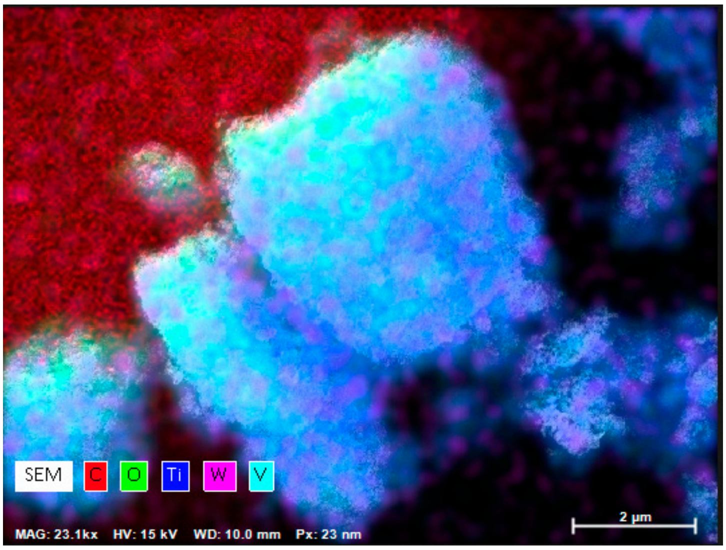

(a)

Map							
Element	Line series	Net counts	Mass Concentration /%	Norm. mass concentration /%	Norm. atomic concentration /%	1σ uncertainty /mass%	1σ rel. uncertainty /%
Carbon	K	167678	50.80	51.49	70.72	0.86	1.69
Oxygen	K	23462	18.61	18.87	19.45	0.40	2.16
Titanium	K	135585	26.45	26.81	9.24	0.61	2.30
Tungsten	L	813	1.37	1.38	0.12	0.14	10.40
Vanadium	K	6223	1.43	1.45	0.47	0.06	4.10
		**Sum**	**98.66**	**100.00**	**100.00**		

(b)

Figure S15. Elemental (a) mapping and (b) quantification of $W_5V_5TiO_2$ calcined at 500°C using EDX. Working distance = 10 mm; Aperture = 60µm ; EHT (accelerating voltage) = 15 kV.

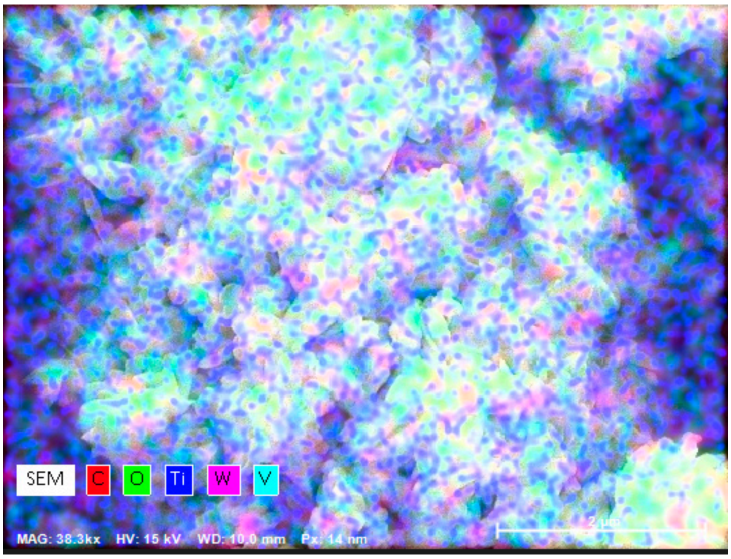

(a)

Map

Element	Line series	Net counts	Mass Concentration /%	Norm. mass concentration /%	Norm. atomic concentration /%	1σ uncertainty /mass%	1σ rel. uncertainty /%
Carbon	K	5477	0.80	0.92	2.03	0.03	3.54
Oxygen	K	133931	34.83	39.82	66.31	0.72	2.06
Titanium	K	731923	47.01	53.75	29.90	0.74	1.57
Tungsten	L	3977	2.61	2.98	0.43	0.13	5.12
Vanadium	K	28555	2.22	2.53	1.32	0.05	2.45
		**Sum**	**87.46**	**100.00**	**100.00**		

(b)

Figure S16. Elemental (a) mapping and (b) quantification of $W_5V_5TiO_2$ calcined at 600°C using EDX. Working distance = 10 mm; Aperture = 60µm ; EHT (accelerating voltage) = 15 kV.

BET Surface Area Analyses

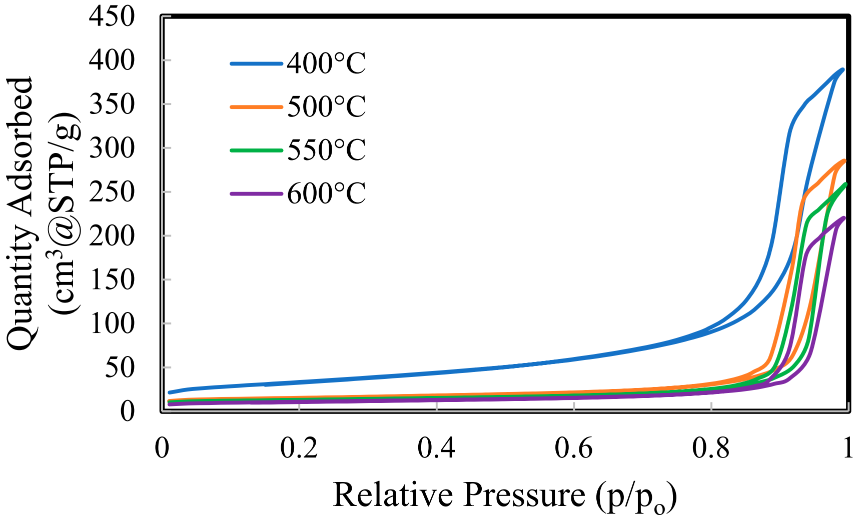

Figure S17. Nitrogen adsorption isotherms for TiO_2

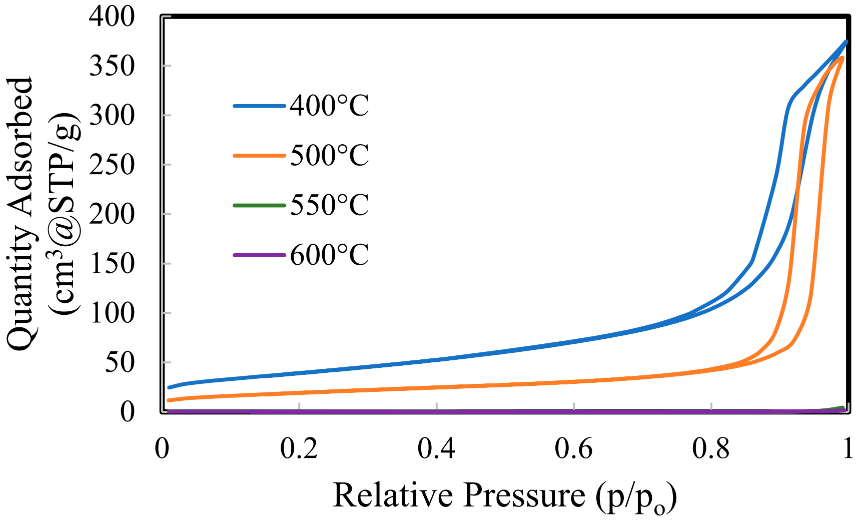

Figure S18. Nitrogen adsorption isotherms for $W_0V_5TiO_2$. The isotherms at 550°C and 600°C are too close to the x-axis to be seen on this graph.

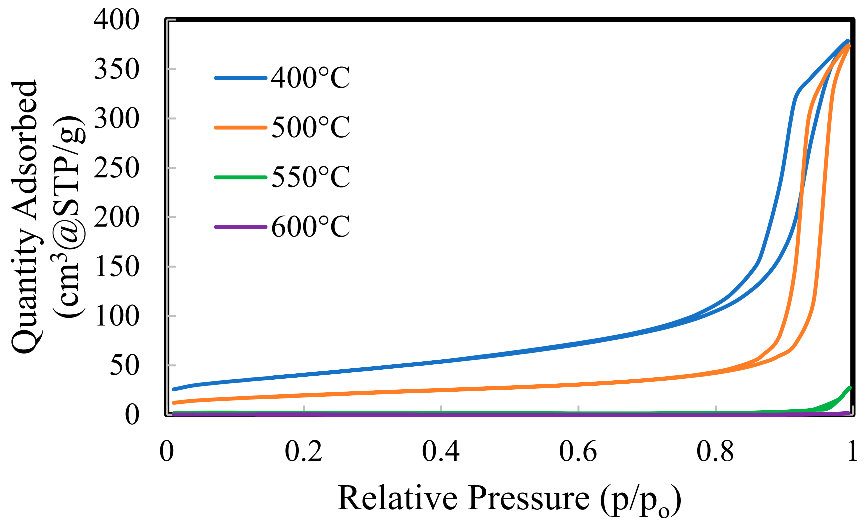

Figure S19. Nitrogen adsorption isotherms for $W_2V_5TiO_2$

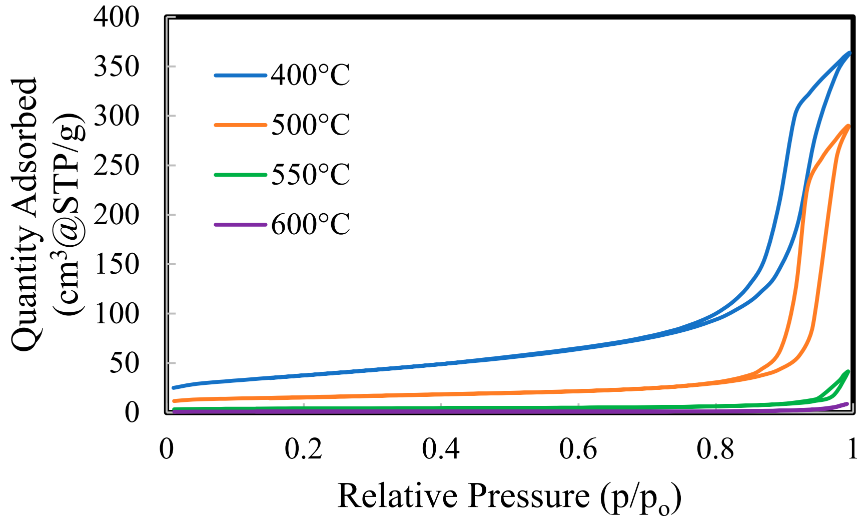

Figure S20. Nitrogen adsorption isotherms for $W_5V_5TiO_2$

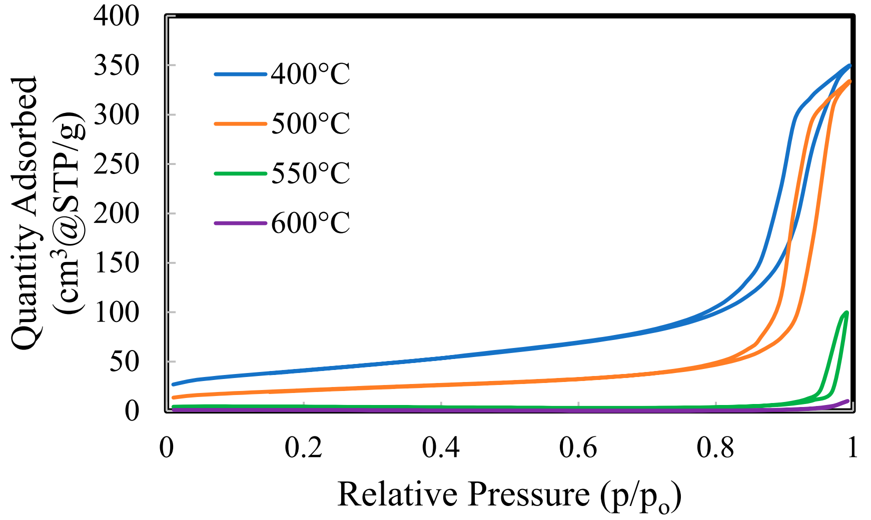

Figure S21. Nitrogen adsorption isotherms for $W_{10}V_5TiO_2$

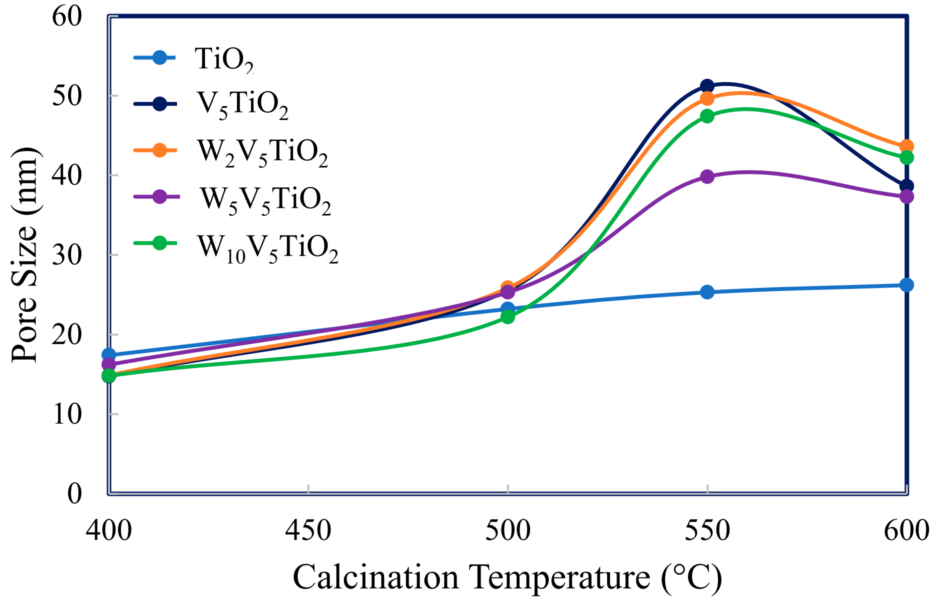

Figure S22. Pore size as a function of calcination temperature and catalyst composition. Pore size determined by nitrogen desorption data and the Barrett-Joyner-Halenda (BJH) model.

Thermal Gravimetric Analyses (TGA) of Catalysts calcined at 400°C.

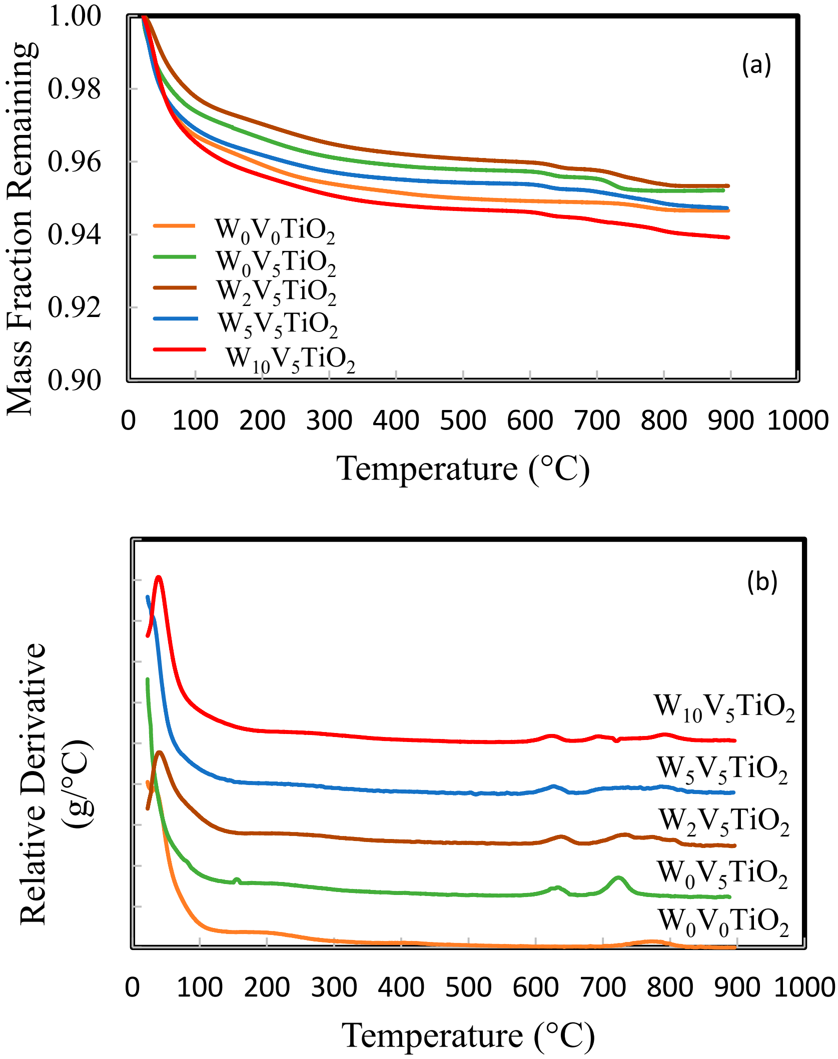

Figure S23. TGA analyses of catalysts that had been calcined in air at 400°C for 24 hours. (a) Mass fraction remaining vs temperature; (b) derivative d(mass)/d(temp) (g/°C)

Table S2. Summary of mass loss as a function of temperature range.

Catalyst ID	< 300°C	300°C to 600°C	> 600°C
$W_0V_0TiO_2$	4.60%	0.48%	0.26%
$W_0V_5TiO_2$	3.84%	0.42%	0.52%
$W_2V_5TiO_2$	3.49%	0.53%	0.64%
$W_5V_5TiO_2$	4.26%	0.35%	0.65%
$W_{10}V_5TiO_2$	4.91%	0.48%	0.70%
